# Advanced and Tailored Nanocomposites for Removing Pollutants from Contaminated Water or Soil Using the Simultaneous “Sense-and-Remove” Strategy: A Review

**DOI:** 10.3390/polym18151892

**Published:** 2026-07-31

**Authors:** Catalina Natalia Yilmaz, Onur Yilmaz, Nona Merry Merpati Mitan, Mihai Brebu, Elena Stoleru, Manoj Karakoti, Doebner von Tumacder, Derya Topkaya Taskiran, Agung Nugroho, Elena Tomsik

**Affiliations:** 1 Biochemistry Division, Department of Chemistry, Faculty of Science, Dokuz Eylül University, Buca, Izmir 35390, Turkey; catalinanatalia.yilmaz@deu.edu.tr (C.N.Y.); derya.topkaya@gmail.com (D.T.T.); 2 Leather Engineering Department, Faculty of Engineering, Ege University, Bornova, Izmir 35100, Turkey; onur.yilmaz@ege.edu.tr; 3 Department of Chemistry, Faculty of Science and Computer Science, Universitas Pertamina, Kebayoran Lama, Jakarta 12220, Indonesia; nona.merry@universitaspertamina.ac.id; 4 “Petru Poni” Institute of Macromolecular Chemistry, 41A Grigore Ghica Voda Alley, 700487 Iasi, Romania; bmihai@icmpp.ro (M.B.);; 5 Institute of Macromolecular Chemistry AS, Heyrovského nám. 2, 162 00 Prague, Czech Republic; karakoti@imc.cas.cz (M.K.); tumacder@imc.cas.cz (D.v.T.); 6 Department of Chemical Engineering, Faculty of Industrial Technology, Universitas Pertamina, Kebayoran Lama, Jakarta 12220, Indonesia; agung.n@universitaspertamina.ac.id

**Keywords:** detection, “sense-and-remove”, water pollutants, soil pollutants, composites, electrochemical detection, bio-waste material

## Abstract

The increasing presence of toxic pollutants in aquatic and terrestrial environments has motivated the need for advanced materials capable of both rapidly detecting and efficiently removing these contaminants. In this review, we summarize recent progress in nanocomposites designed for the simultaneous sensing and remediation of water and soil contaminants, with particular emphasis on heavy metals. We discuss material design strategies, functional components, detection mechanisms, and adsorption performance, highlighting key structure–property relationships. Despite significant advances in sensing and remediation technologies, most existing reviews focus on these functions independently, providing limited discussion of multifunctional materials capable of integrating both processes within a single platform. Our review addresses this gap by critically examining recent developments in advanced nanocomposites for simultaneous pollutant detection and removal, emphasizing material design strategies, structure–property relationships, adsorption and sensing mechanisms, physicochemical characteristics, practical challenges, and future perspectives toward sustainable environmental remediation technologies.

## 1. Introduction

**Scheme 1:**
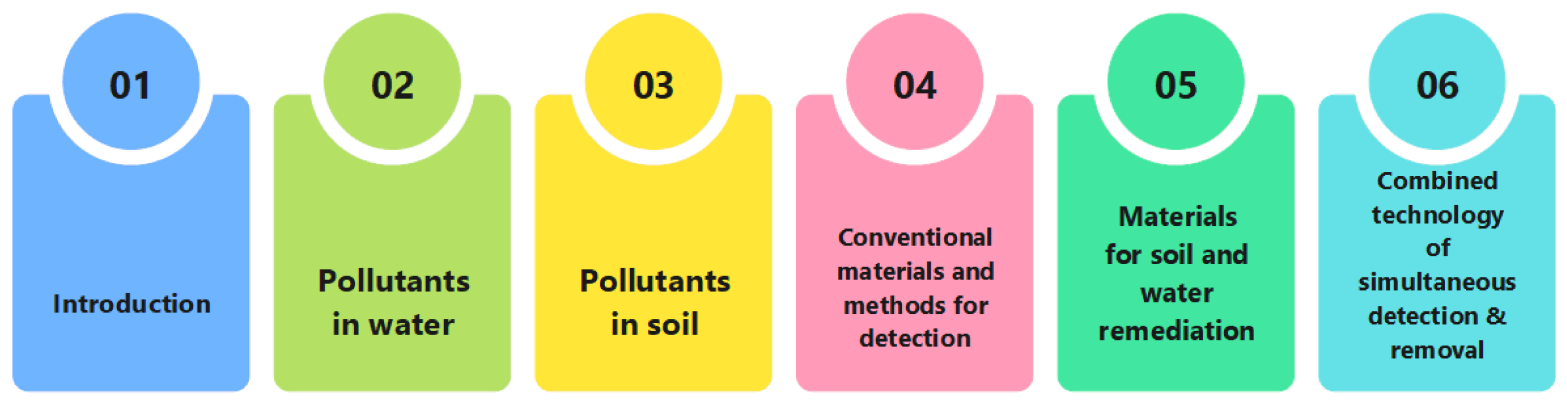
The structure of the review.

The structure of this review follows the outline provided in Scheme 1.

While access to clean water is essential for human health and sustainable development, rapid industrialization, urbanization, population growth, agricultural expansion, and climate change have intensified global water contamination. Although water covers nearly 71% of the Earth’s surface, only a limited fraction of that water is accessible freshwater suitable for human consumption and industrial use. The continuous increase in wastewater generation and pollutant discharge has put pressure on freshwater resources, making water pollution one of the most critical environmental challenges worldwide [1,2,3,4]. In recent decades, increasing industrial and agricultural activities, together with water-related pollution, have exerted tremendous pressure on clean water resources [5,6,7]. According to the United Nations World Water Development report, over 2 billion people reside in countries experiencing water scarcity, while approximately 1.2 billion people lack equitable access to clean drinking water [8]. Contaminants originating from industrial, agricultural, domestic, and commercial activities significantly deteriorate water quality and negatively affect ecosystems, biodiversity, and public health [9,10]. To address these growing concerns, the United Nations Sustainable Development Goals (SDGs) emphasize the urgent need for sustainable water treatment and resource management strategies.

Pollutants are broadly classified into organic chemicals, toxic heavy metals, inorganic non-metallic compounds, and microbial pollutants. Heavy metals, pharmaceuticals, endocrine disruptors, microplastics, persistent organic pollutants, and emerging contaminants such as PFASs are considered hazardous due to their toxicity, persistence, and adverse ecological and health effects [11,12,13]. Heavy metals, released from mining, electroplating, tanning, and textile dyeing [6], are non-biodegradable and may cause neurological disorders, carcinogenicity, renal dysfunction, and developmental abnormalities even at trace concentrations [14,15,16]. Organic pollutants including dyes, pesticides, phenols, pharmaceuticals, personal care products, and endocrine disruptors are also frequently detected in aquatic environments [17,18,19]. Their detection and removal within complex water matrices remain major environmental and technological challenges.

Conventional wastewater treatment technologies, including precipitation, chemical oxidation, solvent extraction, membrane filtration, ion exchange, activated carbon adsorption, reverse osmosis, advanced oxidation processes, and biological treatment systems, have demonstrated effectiveness in removing a variety of contaminants [20]. Among these methods, adsorption is considered one of the most economical, efficient, and facile approaches for wastewater treatment [21]. Nevertheless, conventional treatment systems often suffer from high energy consumption, sludge generation, membrane fouling, operational complexity, secondary pollution, and limited efficiency in treating emerging contaminants [22,23,24,25,26]. Furthermore, most conventional systems are designed either for pollutant removal or detection rather than simultaneous multifunctional operation. These limitations have inspired interest in developing sustainable and advanced treatment technologies capable of integrating efficient sensing and remediation functionalities [27,28].

Beyond conventional treatment processes, extensive research has focused on developing efficient adsorbent and catalytic materials for the removal of heavy metals, dyes, pharmaceuticals, and other emerging contaminants. Conventional adsorbents, including activated carbon, zeolites, clay minerals, silica-based materials, ion exchange resins, and metal oxides, remain among the most widely investigated materials owing to their high adsorption capacity, chemical stability, and well-established removal mechanisms. More recently, sustainable low-cost materials derived from agricultural and industrial bio-waste, such as rice husks, coconut shells, sawdust, sugarcane bagasse, fruit peels, lignocellulosic biomass, biochar, cellulose, and chitosan, have attracted increasing attention due to their abundance, renewability, low production cost, and rich surface chemistry [29]. In parallel, photocatalytic materials, including TiO_2_, ZnO, Fe_3_O_4_, and graphitic carbon nitride (g-C_3_N_4_), have demonstrated excellent degradation efficiencies toward organic pollutants through advanced oxidation processes. Despite these advances, many conventional adsorbents and catalysts exhibit limited selectivity, reduced regeneration efficiency, or decreased performance in complex environmental matrices, emphasizing the need for multifunctional materials capable of integrating pollutant detection with efficient remediation [30].

Soil contamination by pollutants has become a pressing global environmental challenge, driven largely by industrial activities, mining, agriculture, and improper waste disposal. Heavy metals such as lead (Pb^2+^), cadmium (Cd^2+^), zinc (Zn^2+^), and metalloid arsenic (As^3+^) are among the most hazardous soil contaminants, as they persist in the environment, enter the food chain through plant uptake, and pose serious risks to human health. Beyond heavy metals, soils are also threatened by petroleum hydrocarbons, polycyclic aromatic hydrocarbons (PAHs), agrochemicals, and chlorinated solvents, each with distinct chemical behaviors and ecological impacts [31]. Moreover, soil contamination constitutes a direct pathway for groundwater deterioration, as precipitation events facilitate the leaching and downward migration of soil-associated pollutants into underlying aquifers, highlighting the intrinsic interconnection between terrestrial and aquatic contamination.

Addressing these threats requires effective remediation strategies. Conventional approaches include chemical washing, fixation/stabilization, and bioremediation, each with notable limitations: chemical drenching risks secondary pollution and soil structure degradation; stabilization methods may be incomplete or fail over time; and bioremediation, while environmentally friendly, is often too slow for heavily contaminated sites. More advanced methods—such as surfactant-enhanced soil washing, electro-kinetic treatment, and novel adsorbent-based technologies—have gained attention for their improved efficiency and reduced environmental impact. The choice of remediation technology depends on contaminant type and concentration, soil properties, cost, and acceptable timeframes, reflecting the complexity of restoring polluted soils to a safe and productive condition [31].

To overcome the limitations of conventional treatment technologies, nanotechnology has emerged as a transformative approach for water and/or soil purification and environmental remediation. Nanotechnology-based materials possess unique physicochemical properties, including high surface area, tunable surface chemistry, rapid adsorption kinetics, and multifunctional behavior, making them highly promising for both sensing and remediation applications. In recent years, a wide range of adsorbent materials, including activated carbons, clay minerals, carbon nanotubes, zeolites, graphene-based materials, and agricultural waste-derived materials, have been investigated for pollutant removal from polluted systems [32,33]. Advanced nanomaterials, including biochar, biopolymers, clay-based systems, carbon nanostructures, and hybrid nanocomposites, have attracted considerable attention because of their adsorption capacity, environmental compatibility, and structural versatility [34,35,36,37]. In addition to their enhanced adsorption performance, nanomaterials exhibit unique optical, electrical, magnetic, and catalytic properties that further support their application in sensing technologies. Furthermore, their sustainable origin and compatibility with circular economy principles make them good candidates for next-generation environmental technologies [38].

Consequently, increasing attention has been directed toward multifunctional nanocomposites for water and/or soil remediation and monitoring. Combining multiple functional materials within a single nanocomposite platform enables synergistic interactions that improve adsorption capacity, sensing sensitivity, catalytic efficiency, selectivity, and structural stability. Functionalization and hybridization strategies involving metal oxides, conductive polymers, carbon-based nanomaterials, and natural polymers enhance porosity, adsorption performance, and catalytic activity [36,37]. Surface functionalization, heteroatom doping, pore engineering, and magnetic modification are commonly employed to tailor nanocomposite performance toward selective pollutant recognition and enhanced remediation efficiency. Biochar-based composites, chitosan nanocomposites, and hybrid nanostructures have demonstrated significant potential for efficient pollutant removal due to their high adsorption capacity and tunable surface chemistry [39,40]. In particular, stimuli-responsive smart materials capable of responding to pH, temperature, light, or magnetic fields have attracted growing attention due to their tunable selectivity, regeneration capability, and reusability [41]. The reversible and self-cleaning properties of these materials further enhance operational lifetime while reducing secondary pollution and regeneration costs.

Recent advances have enabled the development of multifunctional “**sense-and-remove**” nanocomposites capable of integrating pollutant detection and remediation within a single platform. These systems can simultaneously perform adsorption, photocatalytic degradation, or ion exchange while generating measurable electrochemical, optical, fluorescence, or colorimetric signals during the remediation process. Such integrated platforms enable real-time and on-site water quality monitoring combined with efficient contaminant removal. In particular, sensing platforms based on conductive polymers, metal oxides, biosystems, and carbon nanomaterials have shown enhanced selectivity and sensitivity toward heavy metals and emerging contaminants while simultaneously providing adsorption or catalytic degradation capabilities [35,42]. Moreover, stimuli-responsive materials are designed to dynamically respond to external stimuli for enhancing treatment efficiency and smart operational control [43]. Consequently, multifunctional nanocomposites represent a strategy for next-generation intelligent water treatment technologies.

Despite significant advances in sensing and remediation technologies independently, comprehensive investigations focusing on integrated “**sense-and-remove**” nanocomposite systems remain limited. Most previous reviews have separately discussed pollutant sensing or remediation technologies, while multifunctional nanocomposite platforms integrating both functions remain insufficiently addressed. Additionally, challenges associated with scalability, regeneration efficiency, nanomaterial stability, selectivity, and practical implementation continue to hinder large-scale applications, while potential nanoparticle leaching and long-term ecotoxicological impacts also remain insufficiently investigated. Therefore, a deeper understanding of material design strategies, structure–property relationships, and pollutant interaction mechanisms is necessary for optimizing multifunctional nanocomposites for sustainable water treatment applications.

Although considerable progress has been made in developing materials for pollutant sensing and environmental remediation, the existing review articles have largely addressed these topics independently, focusing either on detection technologies or pollutant removal strategies. Comprehensive analyses integrating multifunctional nanocomposites capable of simultaneously sensing and removing contaminants remain scarce. Furthermore, limited attention has been devoted to correlating material design, physicochemical characteristics, adsorption mechanisms, sensing performance, and practical implementation challenges within a single framework. Therefore, in this review, we aim to bridge these gaps by providing a comprehensive and critical overview of advanced nanocomposites designed for simultaneous “**sense-and-remove**” applications in water and soil remediation. Particular emphasis is placed on conventional and bio-waste-derived materials, structure–property relationships, adsorption and sensing mechanisms, characterization techniques, current limitations, and future research directions toward scalable and sustainable environmental technologies.

## 2. Pollutants in Water

**Scheme 2:**
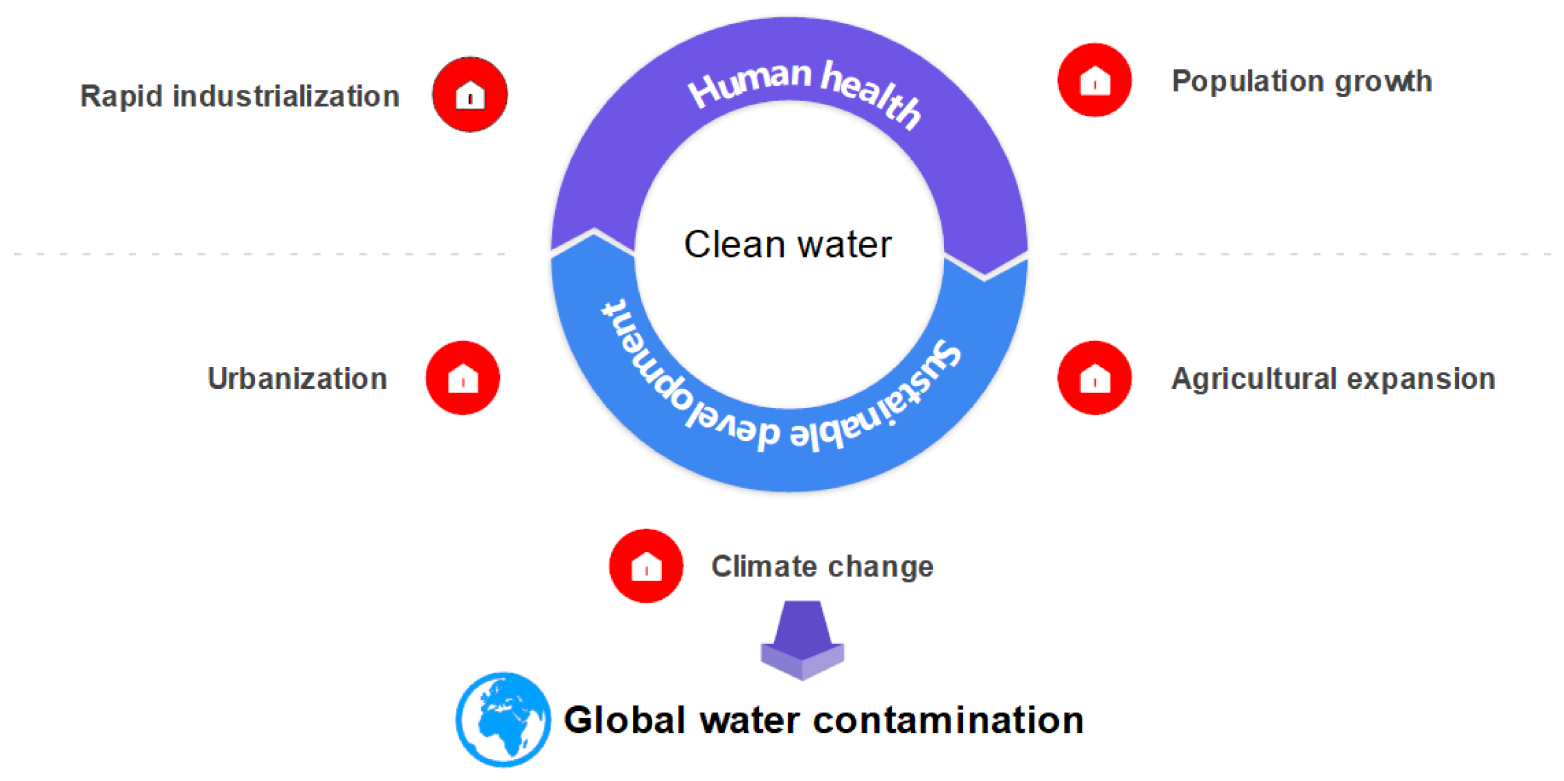
Pollutants in water.

Inorganic pollutants in water are non-biodegradable, non-carbon-based compounds that exist in aqueous environments [44], often originating from industrial, agricultural, or residential sources, as shown in Scheme 2.

Common classifications for inorganic pollutants are shown in Table 1. Based on their chemical nature, inorganic pollutants can be classified as metals and metalloids, inorganic salts (including halides), nutrients, or mineral acids and bases [45]. The complete literature review presented in Table 1 shows the major distribution of each category, their chemical structures, and their pollutant sources.

Metals and metalloids, which are primarily elements that exist as positively charged ions (cations) or complex species, remain central to water quality research due to their toxicity and non-biodegradability. These contaminants are frequently reported in industrial effluents from sources such as electroplating, mining, metal finishing, and electronics.

Inorganic salts represent ions in aqueous systems that increase water salinity and affect osmotic balance. This category includes both the neutral salt and its dissociated ions—for example, sodium chloride (NaCl), fluoride (F^−^), and cyanide (CN^−^). Sources of these pollutants include road salts, seawater intrusion, geological weathering, and mining. Beyond metals, nutrient-type inorganic pollutants such as NO^3−^ and PO_4_^3−^, primarily linked to agricultural fertilizer applications, animal waste, food-processing industries, and domestic sewage, play a crucial role in water contamination.

Mineral acids and bases are chemicals that alter the pH of water, making it corrosive or toxic to aquatic life. Acidic substances containing H_2_SO_4_ and HCl can alter the pH of effluent discharge into the environment, endangering aquatic species. These kinds of pollutants usually come from the mining and chemical industries, which are prevalent in both Southeast Asia (including Indonesia) and Europe, especially in countries with strong chemical and metallurgical sectors such as Germany, Poland, Belgium, the Netherlands, and the Czech Republic.

Table 1 shows the maximum limits for each pollutant category and their effects on humans and the environment, demonstrating that, even at low concentrations, these pollutants can be dangerous.

With the expansion of industry, many efforts have been made to minimize and eliminate pollutants. From a nanocomposite perspective, metal ions are promising targets, as their defined oxidation states and coordination chemistry enable selective adsorption, redox-responsive sensing, and electrochemical detection.

## 3. Pollutants in Soil

### 3.1. Sources and General Characteristics of Soil Pollution

Soil pollution is primarily associated with the accumulation and persistence of hazardous contaminants, including heavy metals, pesticides, petroleum hydrocarbons, radionuclides, and toxic industrial waste [67,68,69,70]. Similarly to water pollution, soil contamination originates from both diffuse and point sources [56], including intensive agricultural practices involving the excessive application of fertilizers, herbicides, and pesticides [71]; industrial activities, mining operations, and petrochemical processes [72]; and improper waste disposal [73,74]. Many soil pollutants are inorganic, non-biodegradable, and strongly associated with mineral phases and organic matter in soil matrices [75,76,77]. Due to the strong physicochemical interactions between soil and water systems, contaminants present in soils may subsequently migrate into groundwater and surface waters through leaching, erosion, runoff, and sediment transport, thereby contributing to the deterioration of water quality and posing risks to ecosystems and human health [78,79]. In addition, volatile compounds and contaminated particulates may be dispersed into the atmosphere through wind erosion [80,81] and volatilization processes [82,83].

### 3.2. Inorganic Pollutants: Heavy Metals and Metalloids

**Scheme 3:**
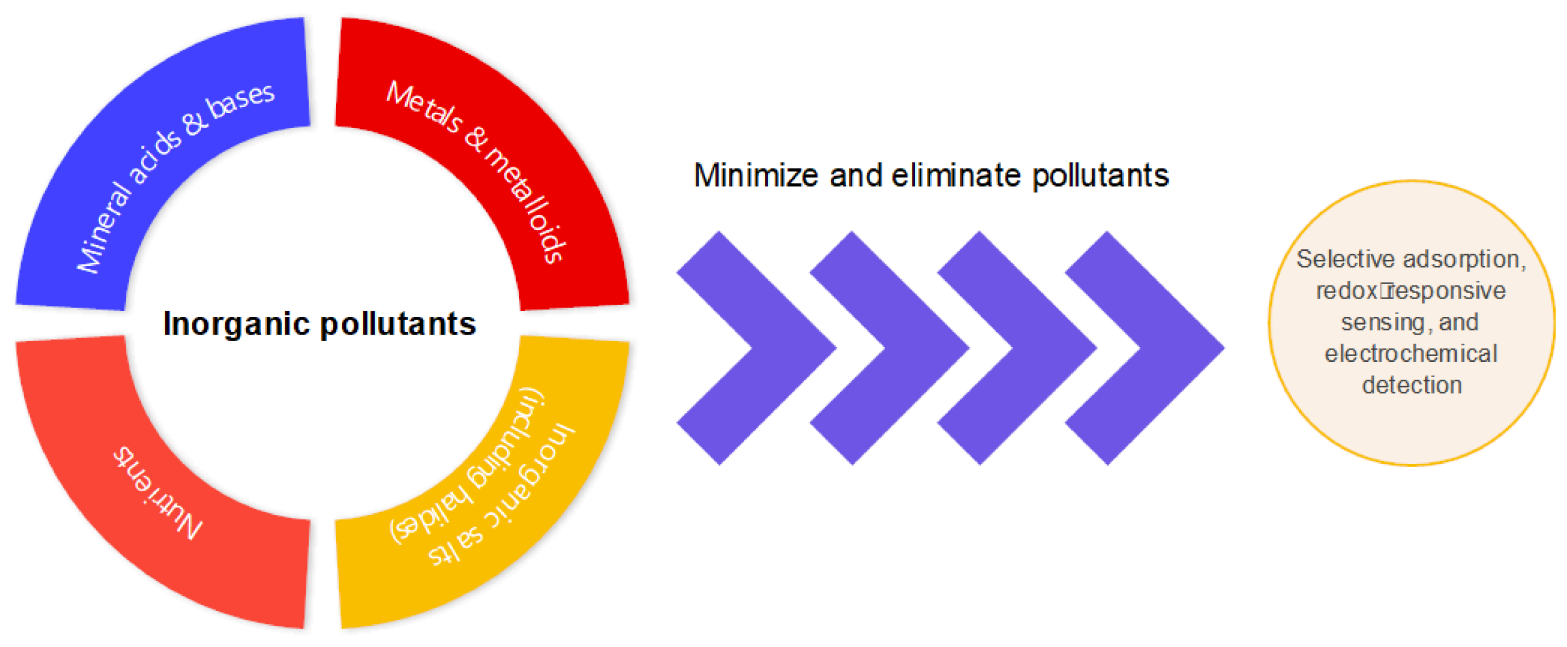
Pollutants in soil.

Particular attention is given to inorganic pollutants, especially heavy metals, as presented in Scheme 3 [84].

Inorganic pollutants arise mainly from mining and smelting, fossil fuel combustion, industrial emissions, battery production, fertilizers, pesticides, sewage sludge, and waste incineration [85,86,87,88]. Natural sources such as volcanic eruptions and erosion also contribute to metal release, although anthropogenic activities are considered the dominant source of contamination [89,90].

**Heavy metals** pose major environmental safety and human health risks because they persist in soils and sediments, bioaccumulate through food chains, enhance the mobility and toxicity of co-contaminants such as pesticides and microplastics, and can ultimately threaten ecosystem stability, biodiversity, crop safety, and human health through long-term exposure and biomagnification [19].

***Their transport through soil, water, and air systems facilitates widespread environmental distribution and the long-term contamination of ecosystems and food resources [91,92].***

### 3.3. Sediment-Bound Contaminants

Unlike soil, which is a dynamic terrestrial system directly supporting plant growth and agricultural productivity, sediments function as depositional sinks in freshwater and marine environments, where contaminants accumulate, persist, and interact under distinct physicochemical and hydrological conditions [93,94].

Sediments play a critical role in the environmental fate of pollutants, acting not only as passive sinks but also as dynamic reservoirs that can release contaminants back into the water column under changing environmental conditions (e.g., contaminated living organisms that decompose and release retained pollutants) [95,96]. Due to sedimentation, adsorption, complexation, and precipitation processes, aquatic bottom sediments accumulate a broad spectrum of contaminants, frequently at concentrations substantially exceeding those in the overlying water column. These contaminated sediments represent long-term environmental risks because they can act as secondary sources of pollution through diffusion, resuspension, redox-driven remobilization, and bioturbation, thereby reintroducing contaminants into aquatic food webs and surrounding waters [97,98,99]. Consequently, understanding the nature, distribution, and behavior of sediment-associated pollutants is essential for assessing environmental risks and for the development of advanced materials capable of both detecting and remediating water contaminants [100].

Compared to soils, where pollutants mainly originate from agricultural inputs, industrial activities, and atmospheric deposition, sediment contamination in aquatic systems is additionally shaped by hydrodynamic transport processes [101]. Major sources of contamination include industrial discharges, agricultural runoff, urban wastewater effluents, and atmospheric deposition, while pollutants transported through the water column frequently adsorb onto suspended particles and accumulate in sediments [102]. As a result, sedimentation processes control the transfer of particle-bound contaminants from the water column to benthic zones [103]. The transfer of contaminants is highly dependent on particle size, surface properties, and hydrodynamic conditions, which complicates the prediction of pollutant behavior in aquatic systems [95]. Moreover, fine-grained sediments enriched in organic matter tend to preferentially accumulate pollutants, leading to persistent and spatially uneven contamination hotspots [104].

The behavior of inorganic pollutants in soils is strongly controlled by soil properties such as pH, redox potential, clay mineral content, and soil organic matter. Acidification, flooding, erosion, and increased temperatures can enhance the mobility and bioavailability of metals, increasing their transfer into groundwater, surface waters, and plants. Flooded or oxygen-deficient soils may promote the release of arsenic and other metals from mineral phases, while elevated temperatures can stimulate transformations of mercury into more toxic forms such as methylmercury. Soil erosion and runoff can further transport metal-contaminated particles to surrounding ecosystems [105].

## 4. Conventional Materials and Methods for Detection

### 4.1. Materials for Pollutant Detection in Water or Soil

Many materials have been investigated for the detection of pollutants, including carbon nanomaterials (CNMs), metal oxide nanoparticles (MOs), metal–organic frameworks (MOFs) and covalent organic frameworks (COFs), conducting polymers (CPs), molecular imprinted polymers (MIPs), and their reciprocal composites. These materials were selected based on their ability to selectively attract pollutants and produce detectable optical, colorimetric, fluorescence, electrochemical, or Raman signals. The following subsections discuss each material class in turn.

#### 4.1.1. Carbon Nanomaterials (CNMs)

CNMs are sensing materials widely used for the detection of heavy metals in contaminated water. They include graphene [106], graphene oxide (GO) [107], reduced graphene oxide (rGO) [108], carbon nanotubes (CNTs) [109], carbon dots (CDs), graphene quantum dots (GQDs) [110], carbon nanofibers (CNFs) [111], and biochar-derived porous carbon (PC) materials [112]. Such materials are commonly employed because of their high surface area, chemical stability, good conductivity, and tunable surface functional groups.

For example, lignin-derived porous carbon (PC) with a layered graphene structure has been used for the detection of Pb^2+^ and Cd^2+^, exhibiting adsorption capacities of 250.5 mg/g and 126.4 mg/g, respectively, along with good regenerative efficiencies of 96% and 92% for Pb^2+^ and Cd^2+^, respectively [112]. Sulfur-doped activated carbon derived from a banana stem-based fluorescent probe was used for the detection of Hg^2+^, exhibiting intense photoluminescence, with a maximum emission at 335 nm upon 230 nm excitation that was selectively quenched by Hg^2+^, even in the presence of multiple interference ions (Ni^2+^, Zn^2+^, Mn^2+^, Fe^3+^, Cr^3+^, K^+^, Ca^2+^, Na^+^, Co^2+^, Cd^2+^, Ag^+^, Pb^2+^, Al^2+^, and Cu^2+^). This high selectively occurred due to the soft–soft binding in Hg^2+^–S interactions. As a result, the sensor exhibited a detection limit of 9.73 µM in real water samples, with a 94–103% recovery [113]. GO and rGO are considered excellent materials for the detection of heavy metals because their oxygen-containing groups, such as –OH, –COOH, and epoxide, can interact with metal ions. Electrochemically reduced rGO-modified glassy carbon electrodes (GCEs) have been used for the simultaneous detection of Pb^2+^ and Cd^2+^ using differential pulse anodic stripping voltammetry [114]. Thiruppathi et al. utilized a fluorinated GO (FGO) for the detection of Cd^2+^, Pb^2+^, Cu^2+^, and Hg^2+^ via square-wave anodic stripping voltammetry (ASV) [57], highlighting that the heteroatom functionalization of GO can improve metal ion sensing without introducing a second inorganic sensing phase. The FGO electrode demonstrated a linear response for Cd^2+^ from 0.6 to 5.0 µM, with a sensitivity of 4.06 µA µM^−1^ and a detection limit of 10 nM. For Pb^2+^, the linear response was 0.3 to 5.0 µM, with a higher sensitivity of 10.32 µA µM^−1^ and a detection limit similar to Pb^2+^. Simultaneous detection was performed over the concentration range of 1.0–6.0 µM for Cd^2+^, Pb^2+^, Cu^2+^, and Hg^2+^. The sensitivities observed during simultaneous detection were 3.64, 6.05, 3.64, and 4.24 µA µM^−1^ for Cd^2+^, Pb^2+^, Cu^2+^, and Hg^2+^, respectively.

CDs and GQDs are often employed in fluorescence-based detection, as their fluorescence can be quenched or enhanced by specific pollutants, allowing for the sensitive detection of metal ions and organic contaminants. For instance, sulfur- and nitrogen-doped CDs have been used as fluorescent probes for detecting Cr^6+^ ions in water [115], exhibiting a detection limit of 16.8 nM. Nitrogen-doped CDs (prepared from highland barley) were used for Hg^2+^ detection [116,117], showing blue fluorescence at 480 nm with a quantum yield of 14.4%. Hg^2+^ quenched the fluorescence of these CDs selectively over other ions, which included Cu^2+^, Cd^2+^, Pb^2+^, Zn^2+^, Mg^2+^, and Ca^2+^. These CD-based sensors demonstrated a linear detection range of 10–160 µM, with a detection limit of 0.48 µM. Similarly, nitrogen-doped CDs (prepared from L-arginine) were studied by Tan et al. (2022) [115] for the parallel fluorescence detection of Cd^2+^ and Hg^2+^. The fluorescence of these CDs was quenched by Cd^2+^ and Hg^2+^, with linear ranges of 0–26.8 µM and 0–49.9 µM, respectively, and detection limits of 0.201 µM (Cd^2+^) and 0.188 µM (Hg^2+^). This sensor was further used in apple and cabbage samples, with recovery values of 86.44–109.40% and 86.62–115.32% for Cd^2+^ and Hg^2+^, respectively. CDs prepared from papaya seed have additionally been used for the detection of Fe^3+^ in an aqueous medium. These CD-based sensors exhibited a selective detection of Fe^3+^ at a low detection limit of 2.35 μM [118]. Nitrogen- and sulfur-co-doped GQDs have been utilized for the detection of Hg^2+^ in water and wastewater [119]. These GQDs had an average particle size of 3.5 ± 0.5 nm, with a quantum yield of 41.9%. The fluorescence intensity decreased proportionally to the Hg^2+^ concentration, resulting in a detection limit of 0.14 nM. Furthermore, this sensor was tested for sewage and dye wastewater samples, showing a linear range of 0.1–15 μM with recoveries of 96–116%.

Carbon nanodots (from beer bagasse) have been used for the development of sensitive fluorometric and electrochemical probes for heavy metal ions. These carbon nanodots exhibited detection limits of 11.3 nM and 78.8 nM for Hg^2+^ and Pb^2+^, respectively, via a fluorometric probe. On the other hand, the electrochemical platform demonstrated a selective and simultaneous detection of metal ions, with detection limits of 124 ng L^−1^ and 551 ng L^−1^ for Hg^2+^ and Pb^2+^, respectively, and a high sensitivity in the range between 11.4 and 34.1 μA nM^−1^ cm^−2^ [120].

Guo et al. (2011) [119] used highly aligned multi-wall carbon nanotube (MWCNT) tower electrodes for the detection of Pb^2+^, Cu^2+^, Cd^2+^, and Zn^2+^ using ASV. The vertically aligned CNT array electrode identified separated stripping peaks for these ions, with detection limits of 12 nM for Pb^2+^, 25 nM for Cd^2+^, 44 nM for Cu^2+^, and 67 nM for Zn^2+^ at a deposition time of 120 s. For this electrode, the detection limit was 0.5 nM for a 10 min deposition. This study was important, as it displayed that the architecture of CNTs alone could improve metal ion detection by ensuring a high surface area and fast electron transfer. Kumar et al. [120] utilized a CNT post electrode for the electrochemical detection of Pb^2+^ and Cu^2+^ in water. This electrode exhibited a linear range of 9.64–168.7 nM for Pb^2+^, with a sensitivity of 0.5095 nA nM^−1^ and a detection limit of 2 nM, equivalent to approximately 0.5 ppb. A similar electrode was also used to detect Cu^2+^, with a detection limit of 41.67 nM (2.64 ppb). The detection limit for Pb^2+^ is lower than the permissible limit set by the World Health Organization for drinking water. It was also found that this electrode could successfully detect Pb^2+^ in the presence of interference ions such as Cd^2+^, Bi^2+^, Mg^2+^, and Hg^2+^, even at concentrations below 5 ppb. A CNT thread electrode was additionally used for the detection of Hg^2+^, Cu^2+^, and Pb^2+^. The calculated detection limits of this electrode were 1.05 nM for Hg^2+^, 0.53 nM for Cu^2+^, and 0.57 nM for Pb^2+^ with a 120 s deposition time [121,122], showing limits of detection comparable to other mercury-free electrodes.

Robinson et al. [122] investigated vertically aligned CNF electrode arrays for Pb^2+^ detection using ASV, achieving a detection limit of 1.73 nM. Zhao et al. [121] similarly used CNFs for the detection of Pb^2+^ and Cd^2+^, showing that the sensitivity for Pb^2+^ detection improved by 8 folds compared to the bare glassy carbon electrode via ASV. This modified electrode exhibited detection limits of 0.9 and 1.5 nm for Pb^2+^ and Cd^2+^, respectively.

Boron-doped diamond electrodes were also examined for the detection of heavy meal ions. The vibrating boron-doped diamond electrode used for the detection of Cd^2+^ via ASV exhibited two linear ranges of 0.01–1 µg L^−1^ and 1–30 µg L^−1^, with a detection limit of 0.04 µg L^−1^ [123]. Similarly, Wu et al. and Kim et al. have reported on boron-doped diamond for the electrochemical detection of Cd^2+^, Pb^2+^, and Cu^2+^ from drinking water [124,125].

#### 4.1.2. Metal Nanoparticles

Beyond carbon-based platforms, noble metal nanoparticles have emerged as another important class of sensing materials due to their unique plasmonic properties. Metal nanoparticles, especially gold (Au) and silver (Ag) nanoparticles (NPs), are commonly used for pollutant detection because of their robust optical and catalytic properties. These NPs exhibit localized surface plasmon resonance (SPR), which changes when the particles aggregate or interact with pollutants, making them useful for colorimetric and surface-enhanced Raman scattering (SERS) detection.

For instance, an AuNP-based colorimetric nanosensor for mercury detection exhibited detection limits of 10 nM and 15 nM for Hg^2+^ and methylmercury, respectively [126,127,128,129]. Citrate-stabilized Ag NPs have been used for the detection of various heavy metals, including Cd^2+^, Cu^2+^, Fe^2+^, Hg^2+^, Mn^2+^, Ni^2+^, Pb^2+^, and Zn^2+^, via the SPR technique [130,131,132,133,134,135,136,137,138,139]. Upon exposure to these metal ions, characteristic shifts in the SPR peak were observed, providing a distinct optical signature for each target ion.

#### 4.1.3. Metal Oxide Nanoparticles (MOs)

Metal oxide nanomaterials, such as TiO_2_, CuO, and NiO, have been used in heavy metal detection due to their high surface area, active adsorption sites, catalytic activity, and improved interfacial electron transfer. TiO_2_ NPs modified on a carbon paste electrode have been used for the separate and simultaneous detection of Hg^2+^, Cu^2+^, and Pb^2+^, exhibiting low detection limits of 15.26, 0.56, and 1.65 nM, respectively. In a ternary solution, the sensor showed an enhanced sensitivity toward Hg^2+^ and Pb^2+^, with detection limits of 8.32 and 0.25 nM, respectively [140]. Similarly, NiO and CuO NPs have been applied for the detection of Cu^2+^ [140], Cd^2+^, Pb^2+^, and Hg^2+^ [141].

#### 4.1.4. Metal–Organic Frameworks (MOFs) and Covalent Organic Frameworks (COFs)

MOFs and COFs are highly porous crystalline materials formed from metal ions or clusters and organic linkers. Their tunable pore size, high surface area, and adjustable chemical functionality make them useful for pollutant and heavy metal detection, and they can interact with heavy metals through coordination, electrostatic attraction, hydrogen bonding, pore size selectivity, and π–π interactions.

A recent report examined a thiacalix[4]arene-based MOF for the electrochemical detection of Cd^2+^ and Pb^2+^, with low detection limits of 0.119 nM and 0.279 nM, respectively [140]. A fully π-conjugated COF, Bpy-sp^2^c-COF, with dual binding sites has been used for the fluorescence recognition and adsorption of Hg^2+^, Ni^2+^, Cu^2+^, and Co^2+^, exhibiting adsorption capacities of 415.34, 165.60, 160.55, and 73.14 mg/g, respectively [141]. These studies demonstrate that MOFs and COFs can act simultaneously as sensing and pollutant-capturing materials.

#### 4.1.5. Conducting Polymers (CPs)

Conducting polymers (CPs), such as polyaniline (PANI), polypyrrole (PPy), and poly(3,4-ethylenedioxythiophene) (PEDOT), are frequently used in the detection of heavy metals because of their electrical conductivity, redox activity, facile electropolymerization, and ability to interact with metal ions through nitrogen- or sulfur-containing functional groups.

For instance, an electropolymerized ion-imprinted polyaniline film was used for selective Ni^2+^ tracking, demonstrating a concentration range of 0.01 to 1 μM and a limit of detection of 0.00482 μM. This sensor also exhibited excellent performance in the presence of interfering ions such as Cd^2+^, Co^2+^, Zn^2+^, and Cu^2+^, even at 1000-fold higher concentrations, and these results were validated against atomic absorption spectroscopy (AAS) in real river water samples (*p* > 0.05, *n* = 3) [131].

PPy-based ion-imprinted and functionalized films have also been used for the selective recognition of heavy metals. Velempini et al. studied a PPy/carboxymethyl cellulose ion-imprinted polymer for electrochemical Hg^2+^ detection in water, exhibiting a detection limit of 0.1 μg/L over a concentration range of 20–800 μg/L [132]. Zhang et al. developed a phytic-acid-functionalized PPy screen-printed sensor for trace Pb^2+^ detection, with a detection range of 10–600 nM and a detection limit of 0.43 nM [133]. A PEDOT/poly(styrenesulfonate) (PSS)-modified GCE was used for the simultaneous detection of Cd^2+^ and Pb^2+^ using linear sweep anodic stripping voltammetry (LSASV), with detection limits of 1.47 μg/mL and 1.15 μg/mL, respectively [134]. N-hydroxyphthalimide-functionalized PEDOT was also used for the voltammetric detection of Hg^2+^, Pb^2+^, and Zn^2+^, showing detection limits of 1.73, 2.33, and 1.99 μg/L, respectively [135].

#### 4.1.6. Molecular Imprinted Polymers (MIPs)

Molecular imprinted polymers (MIPs) represent an important class of synthetic recognition materials that mimic natural receptor binding through the creation of template-shaped cavities within a polymer matrix. These tailor-made recognition sites provide a high selectivity for target metal ions or organic pollutants, even in the presence of structurally similar interferents. MIPs are synthesized by polymerizing functional monomers around a target analyte (the template), which is subsequently removed to leave complementary binding sites. Their combination of selectivity, chemical stability, ease of synthesis, and low cost makes them promising alternatives to biological recognition elements such as antibodies or enzymes.

In the context of heavy metal detection, MIPs are often integrated with electrochemical transducers. For instance, the ion-imprinted PANI and PPy systems described in Section 4.1.5 represent hybrid MIP–conducting polymer architectures in which the imprinting procedure confers ion selectivity, while the electroactive polymer backbone provides the electrochemical signal. Standalone MIP-based sensors have demonstrated selective recognition of Pb^2+^, Cd^2+^, Hg^2+^, and Cu^2+^, and their integration into nanocomposite platforms further enhances their sensitivity and regeneration efficiency.

Nanocomposites combining the material classes described above have been widely studied for heavy metal detection, as they overcome the restrictions of single-component systems. In composites, one component provides strong adsorption and selective binding sites, while another enhances conductivity, catalytic activity, optical response, and mechanical stability. The following paragraphs organize representative examples by composite type.

#### 4.1.7. Carbon/Metal Oxide Composites

Metal oxides can effectively interact with heavy metal ions but may display limited conductivity and low stability; consequently, they are generally combined with conductive carbon nanomaterials such as MWCNTs, rGO, and PC to improve electron transfer, signal response, and stability. MWCNT/ZnO nanocomposites showed a conductivity of 26 S/cm compared to 10^2^ S/cm for ZnO NPs alone, with a lower charge transfer resistance. This translated directly into an improved detection of Cu^2+^, Pb^2+^, and Hg^2+^, with detection limits of 0.8, 1.2, and 0.7 ppb, respectively [142]. Similarly, SnO_2_–MWCNT and SnO_2_–rGO nanocomposites have been utilized for the detection of Pb^2+^, Cd^2+^, Hg^2+^, and Cu^2+^ [143], and Fe_3_O_4_@mesoporous carbon has been used for Pb^2+^ and Hg^2+^ detection [144,145,146]. The MWCNT/CQD/MnO_2_ composite demonstrated electrochemical detection of Cr^6+^ and Cd^2+^, where the carbon phase improves conductivity and MnO_2_ provides active sensing sites, yielding a detection limit of 0.32 μg/L for Cr^6+^ [147].

#### 4.1.8. MOF/Polymer Composites

For the electrochemical sensing of Cd^2+^ and Pb^2+^ in water, including actual Danube River samples, MOF-5/PANI composite electrodes exhibited a higher response for both single and simultaneous detection compared to MOF-5 or PANI alone, with detection limits of 0.077 and 0.033 ppm, respectively [140]. A ZIF-67-derived cobalt/nitrogen-doped carbon (NC)/MWCNT composite exhibited a wider sensing range of 0.12–2.5 μM, with detection limits of 4.5 nM and 4.9 nM for Cd^2+^ and Pb^2+^, respectively [142]. A phytic acid (PA)-functionalized PPy/GO composite-modified GCE was used for the detection of Cd^2+^ and Pb^2+^ via differential pulse voltammetry (DPV), outperforming PA/PPy, PA/GO, and PPy/GO binary systems, with a linear working range of 5–150 μg/L [145].

#### 4.1.9. MXene-Based Composites

A 3D porous melamine-doped rGO/Ti_3_C_2_T_x_ **(MXene) composite-**modified electrode was investigated for the simultaneous detection of Zn^2+^, Cd^2+^, and Pb^2+^. The particle’s 3D structure, based on Ti_3_C_2_T_x_ and rGO sheets, provides a high surface area and developed functional clusters, improving conductivity and metal ion uptake and yielding a sensing range of 3–900 μg/L, with detection limits of 0.48, 0.45, and 0.29 μg/L, respectively [148]. Similarly, a nitrogen-doped carbon-coated Ti_3_C_2_ heterostructure-based electrochemical sensor enabled the simultaneous detection of Cd^2+^ and Pb^2+^ in seawater and tap water, with detection limits of 2.55 nM and 1.10 nM, respectively, and an outstanding selectivity in the presence of interfering ions.

### 4.2. Methods for Pollutant Detection

**Figure 1:**
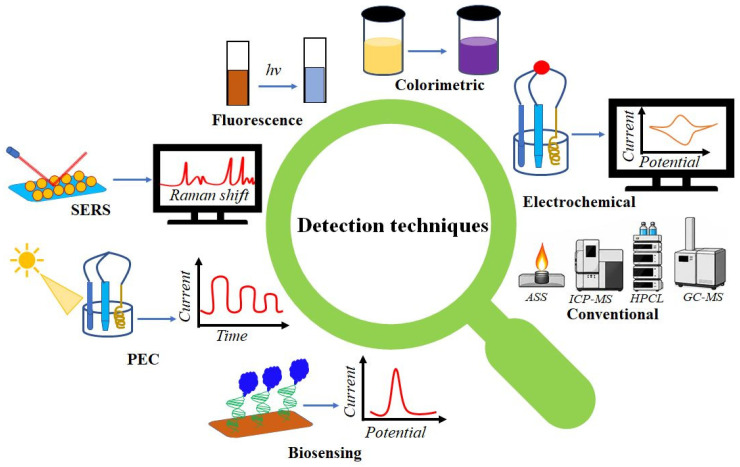
Known methods for the detection of heavy metals.

Many methods for pollutant detection have been developed, including conventional instrumentation, electrochemical methods, colorimetric methods, fluorescence, surface-enhanced Raman scattering (SERS), photoelectrochemical (PEC) methods, and biosensing for the detection of heavy metals in contaminated water (Figure 1 and Table 2).

Conventional instrumental techniques include inductively coupled plasma mass spectrometry (ICP-MS), inductively coupled plasma optical emission spectroscopy (ICP-OES), high-performance liquid chromatography (HPLC), and inductively coupled plasma optical emission spectroscopy (ICP-OES). These techniques are considered reference methods because they provide accurate and validated multi-element quantification at very low concentrations. For example, EPA Method 6020B states that ICP-MS is applicable for determining many elements at sub-μg/L concentrations in water samples, while EPA Method 245.1 reports a working range of 0.2–10 μg/L for mercury determination using cold vapor ICP-OES [144,145]. However, these methods are expensive, laboratory-based, and require trained operators and sample preparation. In contrast, electrochemical methods, including anodic stripping voltammetry, differential pulse voltammetry, square-wave voltammetry, cyclic voltammetry, amperometry, potentiometry, and electrochemical impedance spectroscopy, have been used for the detection of heavy metals. For instance, MWCNT/ZnO nanocomposite-modified electrodes have been utilized for the detection of heavy metals, including Cu^2+^, Pb^2+^, and Hg^2+^, in water samples using electrochemical methods [139]. However, these methods generally suffer due to electrode fouling, pH effects, peak overlap, and interference from co-existing ions.

Colorimetric sensors, often based on Au or Ag NPs, allow for the visual detection of pollutants by changing color due to the interaction between pollutants and sensing probes. These sensors are less quantitative and can be affected by the turbidity or color of the sample. For instance, AuNP-based sensors have been used to detect Hg^2+^ [146]. Fluorescence sensor-based detection measures changes in emission intensity, emission wavelength, or fluorescence lifetime after the interaction between heavy metal ions and a fluorescent probe. This detection process usually occurs via fluorescence quenching, enhancement, ratiometric emission change, electron transfer, inner filter effect, or complex formation. For this, CDs, GQDs, fluorescent MOFs, and organic fluorophores are generally used for the detection of Pb^2+^, Cr^6+^, and Hg^2+^ [147,148,149,150,151]. SERS detects pollutants through Raman signal enhancement near plasmonic nanostructures such as Ag or Au. This is a very promising method because it provides molecular fingerprint information with ultra-low detection limits when combined with chelating molecules, aptamers, or metal-responsive probes [152]. SERS can achieve very low detection limits: for example, a SERS aptasensor detected Hg^2+^ with a detection limit of 0.11 fM [152]. PEC detection combines light excitation with electrochemical information. Semiconductor materials, including TiO_2_, ZnO, CdS, BiVO_4_, Fe_2_O_3_, g-C_3_N_4_, and their composites produce photo-induced electrons and holes under illumination. Whenever metal ions or pollutants interact with the photoactive surface, they change its charge transfer, its recombination, or its surface redox reactions, leading to a photocurrent change. PEC can be used to detect various types of heavy metals and organic pollutants in water samples using various hybrid materials [153].

Biosensing methods use biological recognition elements such as DNA, enzymes, aptamers, antibodies, and peptides to selectively bind metal ions or pollutants. The interaction between recognition elements and heavy metals can be evaluated using electrochemical, fluorescence, colorimetric, SERS, or electrochemiluminescence signals. For instance, DNA and aptamer-functionalized Au NPs have been used for detecting Pb^2+^ and Hg^2+^ in water samples, respectively [154,155,156,157].

To summarize the applied materials and methods a Table 3 is introduced.

## 5. Materials for Soil and Water Remediation

The rapid escalation of environmental pollution in soil and aquatic systems has intensified the demand for efficient, sustainable, and scalable remediation technologies.

### 5.1. Remediation Strategies for Soil

#### 5.1.1. Traditional and Biological Methods

The remediation of contaminated sediments can be approached through a range of strategies. Traditional methods include dredging and physical removal, which directly extract contaminated material but can be disruptive and costly [65]. Alternatively, monitored natural recovery relies on gradual burial and natural attenuation processes. More active in situ approaches include bioremediation techniques, such as bio-stimulation, which enhances indigenous microbial degradation, and bioaugmentation, which introduces specialized microbial strains to accelerate the breakdown of pollutants [66]. In some cases, chemical treatment methods, such as oxidation, are used to transform contaminants into less harmful compounds [65].

#### 5.1.2. Engineered Materials and Advanced Amendments

In recent years, increasing attention has focused on engineered materials and composite amendments for the remediation of metal-contaminated soils and sediments [69]. Materials such as biochar, iron oxides, clay minerals, zeolites, and mineral–organic composites have been applied to reduce the mobility and bioavailability of potentially toxic metal(loid)s through adsorption, ion exchange, surface complexation, precipitation, and redox-mediated stabilization [67,68,69]. These amendments reduce contaminant mobility and porewater concentrations, thereby limiting bioavailability and biological exposure. Advanced nanostructured and reactive hybrid materials are being developed to enhance immobilization efficiency and promote the transformation of contaminants into less mobile or less toxic forms [70]. However, the long-term stability of immobilized metals remains dependent on environmental factors such as pH, redox conditions, and sediment disturbance, which may influence contaminant remobilization over time [71].

### 5.2. Removal Strategy from Water

**Figure 2:**
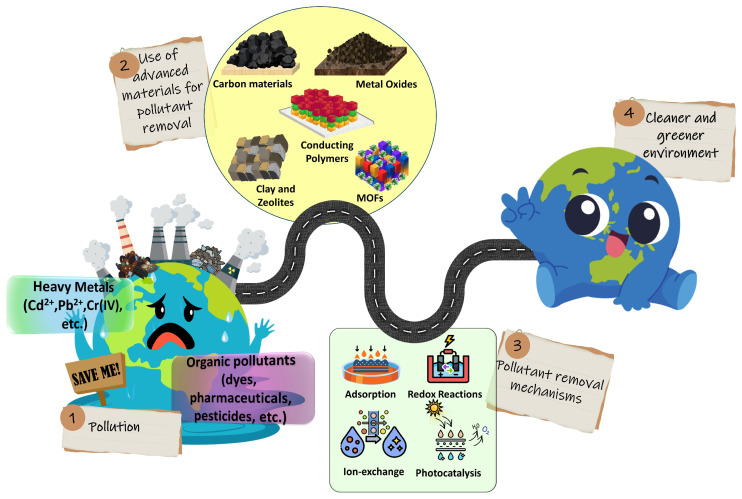
Illustrative roadmap of the use of advanced materials for pollutant removal in soil and water through various mechanisms.

Among the various remediation strategies, material-based approaches—adsorption, photocatalysis, and electro-assisted processes—have emerged as highly effective due to their versatility and tunability. Figure 2 presents a summary of some of the advanced materials used for pollutant removal from soil and water.

#### 5.2.1. Carbon-Based Materials

Carbon-based materials such as biochar [179,180], activated carbon [181], graphene-based composites [182], and carbon nanotubes [183] can effectively remove heavy metals, organic pollutants, pharmaceuticals, pesticides, and per- and polyfluoroalkyl substances (PFASs) from both soil and water via physisorption, π–π stacking, electrostatic interactions, and surface complexation, with performance strongly dependent on feedstock, surface functionalization, and pore structure [157,158]. However, their application in complex matrices is constrained by reduced selectivity, competition among co-existing pollutants, fouling, and matrix effects (e.g., organic matter, pH, clay content), and regeneration often remains difficult or impractical, especially for soil-applied or heavily fouled adsorbents [157,159,160,161].

#### 5.2.2. Metal Oxides

Metal oxides such as Fe_3_O_4_, MnO_2_, and TiO_2_ can remove heavy metals, organics, pharmaceuticals, pesticides, and recalcitrant compounds via adsorption, redox transformation, and photocatalytic degradation [162,163,184,185,186], with surface complexation and ion exchange dominating for metals and reactive oxygen species (ROS)-driven oxidation dominating for organics and pesticides under UV/visible light [187,188]. However, their performance in real soil and water systems is limited by aggregation (reducing accessible surface area), poor colloidal stability, and low light-utilization efficiency, leading to reduced selectivity and incomplete pollutant removal when competing ions, natural organic matter, or variable pH are present [184,189,190].

#### 5.2.3. Natural Materials

Natural materials such as clay minerals (montmorillonite, bentonite) [170,191,192] and zeolites [170,171,172] remove cationic pollutants like heavy metals and ammonium ions mainly through ion exchange and physisorption on their layered or microporous structures, making them suitable for low-cost soil stabilization and groundwater remediation. However, their intrinsic adsorption capacities are generally lower than those of engineered nanomaterials, and their performance is sensitive to pH, competing ions, and matrix composition, so efficiency often drops in complex soil or saline/eutrophic water systems without pre-treatment or modification [173,174].

#### 5.2.4. Polymeric Materials

Polymeric materials, such as PANI and PPy, act as electroactive adsorbents that remove heavy metals, anions, and organic pollutants through electrostatic attraction, ion exchange, and chelation with nitrogen-rich functional groups [175,176,177], while their inherent redox activity and tunable conductivity allow for their integration into electrochemical systems for on-demand regeneration via applied potential [178]. These materials can be fabricated as pure films [178], composites, or porous carbon derivatives [164,165], enhancing surface area and interaction strength with pollutants, but their performance is still limited by their stability under repeated cycling, sensitivity to pH and matrix composition, and lower intrinsic capacity compared with some advanced nanomaterials [166,167,168].

#### 5.2.5. MOFs

MOFs achieve a highly selective adsorption and catalytic degradation of heavy metals and organic micropollutants by exploiting their exceptional surface area, tunable pore geometry, and designable functional groups, which allow for strong interactions such as coordination, electrostatic attraction, π–π stacking, and host–guest inclusion [163,193,194,195]. Nevertheless, their practical deployment in soil and water remediation is constrained by their limited water stability (hydrolysis of metal-linked bonds), high synthesis and purification costs, and difficulties in scaling up production while maintaining performance, which together hinder broad industrial adoption despite their impressive lab-scale capacities [196,197,198].

#### 5.2.6. Hybrid Electrochemical Remediation Systems

Recent advances in hybrid electrochemical remediation combine adsorption, redox activity, and conductivity to improve the removal of organic pollutants and nutrients from water. Carbon-supported metal oxides and conducting polymer-based electrodes are used in systems such as capacitive deionization (CDI) [199,200,201], hybrid electrochemical–granular activated carbon treatment [202], and electrocoagulation-based reactors, where an electric field drives pollutant uptake and later enables controllable regeneration [203,204]. These approaches also reduce secondary waste generation because pollutants can be desorbed, transformed, or recovered instead of being locked into spent sorbents, and some systems pair removal with resource recovery, such as ammonia capture. Their main constraints are still electrode fouling, conductivity loss, aggregation, and operating-condition sensitivity, so performance depends strongly on current density, electrolyte composition, and the nature of the wastewater matrix [205,206,207,208,209,210].

## 6. Combined Technology of Simultaneous Detection and Removal

Traditional water or soil treatment relies on separate systems for detecting pollutants and cleaning them up. This split leads to slow analysis, higher operational costs, and delayed responses to environmental hazards. To fix this, researchers are turning to multifunctional materials that detect and remove contaminants at the same time. Combining sensing with adsorption or degradation within a single structure offers the potential for real-time monitoring and instant treatment. Coupled sensing techniques for heavy metal detection, when integrated into advanced multifunctional platforms like hydrogels, rely on combining dynamic physical, chemical, or biological recognition elements with a fast signal transducer. Instead of just trapping the metal or sending a delayed sample to a lab, these systems output an immediate, quantifiable signal upon binding [189].

In multifunctional “**sense-and-remove**” platforms, sensing is directly integrated with simultaneous contaminant adsorption or degradation, enabling real-time monitoring and purification within a single material system. Coupled sensing techniques for heavy metal detection have been classified into electrochemical, optical, and biological transduction pathways [190]. Electrochemical sensing is highly favored for its trace-level detection [191]. In the anodic stripping voltammetry (ASV) detection method, heavy metal ions undergo a two-step process consisting of preconcentration—where they are adsorbed, reduced, and deposited onto a conductive surface at a negative potential—followed by stripping [191]. This electrical signal can be amplified by incorporating advanced materials like graphene or bismuth nanoparticles into the matrix, which form alloys with target metals to considerably reduce detection limits [192].

Optical and spectroscopic coupled techniques rely on changes in light absorption, emission, or propagation within the material architecture. These can occur through fluorescence quenching or enhancement, where heavy metals act as electron acceptors to trigger photo-induced electron transfer (PET) or the heavy atom effect, shifting the brightness of embedded fluorophores or quantum dots [170]. To mitigate environmental background noise, ratiometric fluorescence utilizes dual-emission fluorophores to calibrate precision based on the intensity ratio of two wavelengths, while solid hybrid sensing strips utilize carbon dots embedded in biopolymer hydrogels like agarose or chitosan to enable the simultaneous colorimetric visual detection and filtration-driven separation of metals like Cr^6+^, Cu^2+^, and Pb^2+^ [171].

Bio-coupled sensing addresses the historical challenge of cross-interference by integrating highly specific biological recognition elements into the system framework [190]. This includes aptamer-coupled sensors, where synthetic single-stranded DNA or RNA oligonucleotides undergo dramatic conformational folding upon binding specific ions like mercury, mechanically pulling signal tags closer to the transducer surface [170]. Similarly, enzymatic inhibition strategies exploit the natural toxicity of heavy metals by embedding enzymes like glucose oxidase or urease into electroactive hydrogels; the presence of contaminants disrupts enzymatic activity, allowing researchers to monitor pollution levels in real-time through localized variations in bioelectric current, pH drops, or reductions in byproduct formation [172].

The rapid development of multifunctional remediation materials has resulted in a wide variety of coupled sensing–remediation platforms employing optical, electrochemical, biological, magnetic, and catalytic transduction mechanisms. A comparative overview of the major coupled sensing–remediation technologies currently utilized for simultaneous pollutant monitoring and removal is presented in Table 4.

As summarized in Table 4, fluorescence-based hydrogels remain among the most extensively investigated systems due to their rapid response, portability, and high sensitivity. In contrast, electrochemical platforms generally provide superior quantitative accuracy and ultra-low detection limits, while catalytic and self-reporting systems offer autonomous remediation and real-time monitoring. Nevertheless, issues related to signal interference, regeneration efficiency, long-term stability, and large-scale applicability continue to limit the practical implementation of such materials [173].

An emerging concept in multifunctional hydrogel systems is the development of “self-reporting” remediation platforms, where pollutant adsorption and sensing occur simultaneously within the same material matrix. These multifunctional hydrogels operate through fluorescence quenching, colorimetric or photonic shifts, and shape-memory deformation induced by heavy metal binding within the polymer network. Such dynamic responses enable simultaneous contaminant adsorption and real-time visual or analytical monitoring [174,175].

The multifunctionality of hydrogel-based sensing platforms is primarily governed by the physicochemical interactions occurring within the polymer network and at the hydrogel–pollutant interface.

### 6.1. Fundamental Mechanisms of “Sense-and-Remove”

Hydrogels are three-dimensional polymeric networks capable of retaining large amounts of water while providing abundant pore structures, large specific surface areas, and diverse hydrophilic functional groups, such as –COOH, –NH_2_, –OH, –SO_3_H, and –CONH_2_ [164,165,166,176,177,178]. These functionalities can strongly interact with heavy metal ions through electrostatic attraction, chemical chelation, ion exchange, and hydrogen bonding, enabling efficient pollutant adsorption and removal. Beyond adsorption, fluorescent nanomaterials, including carbon dots (CDs), metallic nanoclusters, and quantum dots, can be incorporated into hydrogel matrices to impart sensitive sensing capabilities [167,168,169,193,194]. These fluorescent hydrogels act as adsorption aggregators, concentrating heavy metal ions within the hydrogel network and promoting fluorescence quenching through non-radiative energy transfer or metallophilic interactions, thereby enabling simultaneous pollutant detection and removal.

### 6.2. Fluorescence-Based Hydrogel Sensing Platforms

Nanosized fluorescent sensors such as carbon dots (CDs) and metallic nanoclusters possess abundant active sites that can selectively interact with heavy metal ions, resulting in significant fluorescence quenching. Compared with conventional chemo-sensors, these fluorescent nanomaterials exhibit excellent optical properties, superior hydrophilicity, low toxicity, facile synthesis, and high photostability [193,194]. Incorporating fluorescent probes into hydrogel matrices enables the development of multifunctional materials capable of both pollutant adsorption and highly sensitive optical detection. Importantly, the localized enrichment of heavy metal ions within the hydrogel network significantly amplifies the sensing response.

To overcome the limitations of dispersion and recovery, recent studies have focused on embedding fluorescent MOFs into hydrogel matrices to create stable multifunctional sensing platforms [175,195]. He et al. developed an SA/PAM@MOF-Eu hydrogel by incorporating Eu-based MOFs into sodium alginate/acrylamide hydrogels, enabling the selective fluorescence detection of Co^2+^, Cu^2+^, and Ni^2+^ with detection limits of 0.18, 0.08, and 0.12 μM, respectively. The incorporation of 2,6-pyridine dicarboxylic acid improved MOF dispersion and stability within the polymer network, resulting in enhanced water stability, portability, and mechanical durability. These multifunctional hydrogel systems demonstrate a strong potential for practical real-time heavy metal detection.

### 6.3. Electrochemical Detection–Removal Platforms

Electrochemical detection–removal platforms have appeared as multifunctional systems. These platforms integrate contaminant adsorption with electrochemical signal transduction, enabling the rapid, sensitive, and real-time detection of toxic metal ions during the remediation process.

Voltammetry techniques, including anodic stripping voltammetry (ASV), differential pulse voltammetry (DPV), square-wave voltammetry (SWV), and electrochemical impedance spectroscopy (EIS), are widely employed methods due to their high sensitivity, portability, and ultra-low detection limits. Recent studies have demonstrated that the incorporation of nanomaterials such as graphene, reduced graphene oxide (rGO), carbon nanotubes (CNTs), MXenes, metal nanoparticles, and metal–organic frameworks (MOFs) enhance electron transfer efficiency, catalytic activity, and electrochemical signal amplification, as discussed in Section 4 [196]. Furthermore, innovative sensing strategies including ion-imprinted polymers (IIPs), flexible wearable sensors, paper-based electrochemical platforms, dual-mode electrochemical–fluorescence systems, and microfluidic electrochemical devices have considerably improved selectivity, portability, anti-interference capability, and field applicability.

**Figure 3:**
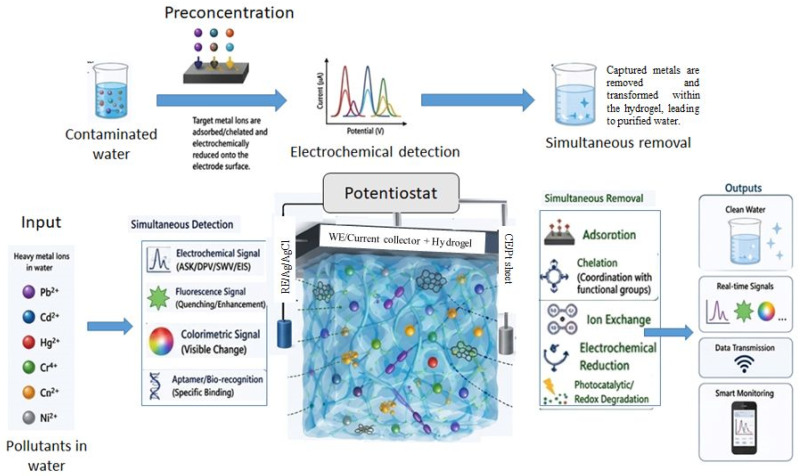
Schematic of electrochemical detection–removal mechanisms in multifunctional hydrogel systems (part of the figure was generated using AI Gemini).

Compared with fluorescence-based sensing systems, electrochemical platforms generally provide lower detection limits, higher quantitative precision, and easier integration into portable electronic devices; however, they remain more susceptible to electrode fouling and signal instability in complex environmental matrices. The multifunctional operation principles of electrochemical hydrogel-based detection–removal systems are schematically illustrated in Figure 3.

In multifunctional electrochemical hydrogel platforms, heavy metal ions are preconcentrated inside conductive hydrogel matrices through chelation, ion exchange, and electrostatic interactions, followed by electrochemical signal detection using one of the following techniques—anodic stripping voltammetry (ASV), differential pulse voltammetry (DPV), square-wave voltammetry (SWV), or electrochemical impedance spectroscopy (EIS). Simultaneously, adsorption, electrocatalytic reduction, and photocatalytic degradation pathways contribute to pollutant removal.

Recent studies have focused on improving the electrode interface. Abilkas et al. [197] evaluated the critical role of material interface modification in upgrading glassy carbon electrodes (GCEs) for simultaneous multi-ion heavy metal tracking. The study reported that achieving trace-level sensitivity for co-existing ions like cadmium, lead, mercury, and chromium depends on fine-tuning interfacial coordination chemistry, charge transfer kinetics, and stripping equilibria. The authors demonstrated that modifying carbon substrates with metallic nanoparticles creates active sites that promote efficient metal nucleation and beneficial alloy formation. Shahzad et al. additionally reported that hydrogel-based materials have emerged as multifunctional platforms for both the adsorption/removal and electrochemical detection of heavy metal pollutants. They demonstrated that hydrogels containing –OH, –NH_2_, –COOH, and –SO_3_H groups exhibit excellent adsorption capacities toward toxic metal ions through mechanisms such as chelation, ion exchange, electrostatic attraction, and coordination interactions [189].

Lv et al. reported a novel combined sampling–mass spectrometry platform for the simultaneous online detection of volatile organic compounds (VOCs) and heavy metal ions in water using vacuum ultraviolet photoionization time-of-flight mass spectrometry (VUP-TOF-MS) [198]. The developed system integrated spray-based VOC extraction with hydride generation chemistry to convert dissolved heavy metal ions into volatile metal hydrides prior to detection. By combining photon ionization (PI) and photoelectron chemical ionization (PCI) modes within a single analytical platform, the method enabled rapid switching between VOC and heavy metal detection, with response times generally below 40 s. The system demonstrated high sensitivity, good repeatability, and satisfactory recoveries (94–118%) for real water samples, while achieving detection limits ranging from sub-μg L^−1^ levels for VOCs to low μg L^−1^ levels for heavy metal ions.

Beyond electrochemical sensing, several multifunctional platforms simultaneously utilize electro-assisted adsorption [199], electrosorption [200], electrocoagulation [201,202], and electrocatalytic reduction processes for active pollutant removal [203,204].

Despite their excellent sensitivity and analytical performance, electrochemical **“sense-and-remove”** systems still face several technical and operational limitations that restrict their large-scale application. (1) Electrode fouling and passivation [205] caused by natural organic matter, (2) mineral scaling, and biofilm accumulation [206] can significantly reduce electron transfer efficiency, and (3) block active catalytic sites over prolonged operation [207]. Complex environmental matrices may interfere with adsorption equilibria and electrochemical signal generation, leading to reduced selectivity, overlapping stripping peaks, and signal instability. Furthermore, parasitic side reactions such as hydrogen and oxygen evolution may decrease energy efficiency, while catalyst poisoning, nanoparticle delamination, and material degradation can negatively affect long-term operational stability. Therefore, future research should prioritize antifouling electrode architectures, durable multifunctional nanocomposites, energy-efficient reactor configurations, and scalable continuous-flow electrochemical remediation systems for real-world applications [214].

### 6.4. “Sense-and-Remove” from Water

Representative combinations of fluorescent materials and hydrogel matrices that have been utilized for simultaneous pollutant detection and removal are summarized in Table 5.

Fluorescence-based optical sensing is the most widely utilized detection strategy in multifunctional hydrogel systems, where fluorescence quenching or signal shifts occur upon heavy metal ions binding to embedded carbon dots (CDs), carbon nanodots (CNDs), or quantum dots within the hydrogel matrix. These sensing platforms are generally rapid, cost-effective, and portable. Simultaneously, heavy metal adsorption is primarily governed by chelation, ion exchange, and electrostatic interactions provided by functional groups such as –NH_2_, –COOH, and –OH. The adsorption performance of representative hydrogel systems is summarized in Table 6.

Despite the adsorption capacities and sensing performances reported for multifunctional hydrogel systems, many of these systems remain limited to laboratory-scale conditions using simplified aqueous solutions, and real wastewater matrices containing competing ions, organic matter, and fluctuating pH conditions may significantly affect their sensing accuracy and adsorption efficiency.

Although several multifunctional hydrogel systems have demonstrated acceptable reusability over multiple adsorption–desorption cycles, further improvements in mechanical stability, regeneration efficiency, and long-term operational performance are still required for large-scale environmental deployment.

### 6.5. “Sense-and-Remove” from Soil

Surfactant-enhanced soil remediation (SESR), reviewed comprehensively by Tiwari and Tripathy [223], provides a complementary framework in which amphiphilic molecules simultaneously mobilize contaminants for extraction and generate measurable elution signals. The progressive appearance of metal–surfactant complexes in the eluate mirrors the depletion of metals in the solid phase, providing a real-time concentration profile that functions as a detection signal integrated within the remediation workflow.

Electro-kinetic remediation combined with biosurfactants pushes this concept further. When rhamnolipid and polyaspartic acid (PASP) were applied alongside an electric field, their removal efficiencies reached 66% for Cu, 67.1% for Ni, 61.2% for Cr, and 61.8% for Pb. The directional migration of metal ions under the applied potential is inherently quantifiable, enabling continuous monitoring of flux—a direct proxy for the real-time detection of contaminant mobilization.

### 6.6. Water Filtration Technologies Using Conventional and Bio-Waste-Derived Materials

Membrane-based water filtration technologies have become an important component of modern water treatment due to their high separation efficiency and compatibility with continuous operation. Conventional filtration systems, including microfiltration (MF), ultrafiltration (UF), nanofiltration (NF), and reverse osmosis (RO), are widely employed for the removal of suspended solids, microorganisms, heavy metals, dyes, pharmaceuticals, and other emerging contaminants. However, membrane fouling, high energy consumption, and operational costs remain significant challenges that limit their long-term application [224]. To improve sustainability, increasing attention has been directed toward filtration materials derived from renewable bio-waste resources. Cellulose, chitosan, lignin, biochar, agricultural residues, and electrospun biopolymer membranes have demonstrated promising filtration performance due to their biodegradability, abundant surface functional groups, and low production costs [225]. These multifunctional filtration systems combine size exclusion with adsorption and catalytic degradation, making them promising candidates for integrated sense-and-remove applications in sustainable water treatment.

### 6.7. Adsorption Kinetics, Thermodynamics, and Mechanistic Interpretation

Adsorption kinetics and thermodynamic analyses provide valuable insight into the mechanisms governing pollutant removal by multifunctional nanocomposites. The pseudo-first-order (PFO) model is generally associated with physisorption, whereas the pseudo-second-order (PSO) model often indicates chemisorption involving electron sharing or exchange between adsorbent functional groups and pollutant molecules [226]. However, adsorption mechanisms should not be inferred solely from kinetic fitting, as multiple interactions may occur simultaneously depending on the physicochemical properties of both the adsorbent and the contaminant.

Thermodynamic parameters further complement mechanistic interpretation. Negative Gibbs free energy (ΔG°) values indicate spontaneous adsorption, while the enthalpy change (ΔH°) distinguishes endothermic from exothermic processes and provides insight into the strength of adsorbent–adsorbate interactions. In addition, positive entropy changes (ΔS°) suggest increased randomness at the solid–liquid interface during adsorption. Combined with adsorption isotherms and characterization techniques such as FTIR and XPS, these analyses help distinguish between physisorption, chemisorption, ion exchange, electrostatic attraction, hydrogen bonding, and surface complexation, thereby providing a more comprehensive understanding of pollutant removal pathways and supporting the rational design of multifunctional sense-and-remove materials [227].

### 6.8. Influence of Porosity and Surface Characteristics on Sense-and-Remove Performance

The efficiency of multifunctional nanocomposites for simultaneous pollutant detection and removal is strongly influenced by their structural properties, particularly their specific surface area, pore volume, and pore size distribution. These characteristics determine the accessibility of active adsorption sites, facilitate pollutant diffusion, and influence the interaction between contaminants and the material surface. Brunauer–Emmett–Teller (BET) analysis is widely employed to determine the specific surface area of porous materials, whereas pore volume and pore size distribution are commonly evaluated using the Barrett–Joyner–Halenda (BJH) method. Materials with high surface areas and interconnected pore networks generally exhibit superior adsorption capacities and faster mass transfer, contributing to improved removal efficiency. Representative BET surface areas of commonly used conventional and bio-waste-derived materials are summarized in Table 7.

As shown in Table 6, conventional materials generally exhibit higher specific surface areas than untreated bio-waste-derived materials. Nevertheless, activation and functionalization strategies can significantly enhance the pore structure and surface chemistry of biomass-derived adsorbents, enabling adsorption performances comparable to those of conventional materials while providing additional sustainability advantages. It should also be emphasized that adsorption efficiency is not governed solely by the BET surface area. Surface functional groups, pore accessibility, chemical affinity, and pollutant–adsorbent interactions collectively determine the overall performance of multifunctional nanocomposites. Therefore, optimizing both pore architecture and surface chemistry is essential for the rational design of advanced sense-and-remove materials capable of achieving high adsorption efficiency together with rapid and sensitive pollutant detection.

A comprehensive evaluation of the structural and surface properties of conventional and bio-waste-derived materials requires the use of complementary physicochemical characterization techniques. Scanning electron microscopy (SEM) and transmission electron microscopy (TEM) are commonly employed to investigate surface morphology, particle size, pore architecture, and nanoparticle dispersion, whereas atomic force microscopy (AFM) provides high-resolution information on surface roughness and topography. X-ray diffraction (XRD) is widely used to determine crystalline phases and structural order, while Raman spectroscopy is particularly valuable for evaluating graphitic structures and defect density in carbon-based materials. Surface chemistry and elemental composition are typically analyzed using X-ray photoelectron spectroscopy (XPS) and Fourier-transform infrared spectroscopy (FTIR), whereas solid-state ^13^C NMR provides detailed information on the carbon framework and functional groups of bio-based materials. Together with BET analysis, these complementary techniques establish the structure–property relationships governing adsorption capacity, sensing performance, and the overall efficiency of multifunctional sense-and-remove nanocomposites.

## 7. Scope for Future Research

### 7.1. Bio-Waste-Derived Materials for “Sense-and-Remove” Strategy

The valorization of agricultural and industrial bio-waste into functional nanocomposites represents one of the directions for sustainable water treatment. Lignocellulosic residues—including rice husks, sugarcane bagasse, corn cobs, orange peels, coffee grounds, and spent tea leaves—are abundant, low in cost, and chemically rich in hydroxyl, carboxyl, and amino groups that can be directly exploited for pollutant chelation and sensing signal transduction. The conversion of these feedstocks into engineered biochar, activated carbon, carbon dots (CDs), and cellulose nanofibers via pyrolysis, hydrothermal carbonization, or chemical activation yields materials with tunable surface chemistry, high surface area, and intrinsic fluorescence—properties ideally suited for “**sense-and-remove**” platforms.

Future research should prioritize the development of bio-waste-derived carbon dot composites that retain fluorescence activity after being embedded within hydrogel or membrane matrices, enabling the simultaneous optical detection and adsorption of heavy metals. The demonstrated capacity of lignin-derived fluorescent hydrogels for Cr^6+^ detection and removal (599.9 mg/g) confirms the feasibility of this approach, yet the range of target pollutants and bio-waste feedstocks explored remains narrow. Expanding to PFASs, pharmaceuticals, and microplastics—using tailored surface functionalization strategies—would significantly broaden the applicability of this method.

Chitosan and cellulose obtained from bio-waste streams (shrimp shells, agricultural residues) offer promising scaffolds for multifunctional nanocomposites. Their abundant –NH_2_ and –OH groups enable metal ion coordination, while chemical crosslinking and hybridization with graphene oxide or conductive polymers can introduce electrochemical activity. Waste cotton-derived chitosan/EDTA biochar composites have already demonstrated exceptional Pb^2+^ and Cu^2+^ adsorption (>1100 mg/g), and the further integration of electroactive components into such systems could enable the simultaneous electrochemical quantification of adsorbed ions.

Circular economy alignment is a critical driver: bio-waste-derived materials should be designed with end-of-life scenarios in mind, including thermal regeneration, magnetic recovery, or biodegradation pathways that minimize secondary contamination. Life cycle assessment (LCA) and techno-economic analysis should accompany material development to bridge the gap between laboratory performance and scalable deployment.

### 7.2. Electrochemical Methods for Real-Time and On-Site Detection

Electrochemical sensing represents the most translatable detection platform for integrated “sense-and-remove” systems due to its inherent compatibility with conductive nanocomposites, low instrumentation cost, miniaturization potential, and quantitative response. Future progress should focus on three key directions: multianalyte simultaneous detection, coupling with remediation workflows, and field-deployable miniaturized systems.

Multianalyte electrochemical sensors capable of resolving overlapping stripping peaks of Pb^2+^, Cd^2+^, Cu^2+^, Zn^2+^, Hg^2+^, and As^3+^ in a single measurement remain a major analytical challenge. New composite electrode materials—such as Mxene/rGO aerogels, nitrogen-doped carbon/Ti_3_C_2_ heterostructures, and MOF-derived carbon/MWCNT hybrids—have demonstrated simultaneous multi-metal detection at sub-nanomolar limits of detection with excellent selectivity. Moreover, it has recently been shown that it is possible to obtain a selective polymer film for Pb^2+^ detection using acid-assisted polymerization, even with high concentrations of interfering ions [236]. Incorporating bio-waste-derived carbon into these architectures could reduce cost and improve biocompatibility while maintaining high conductivity and active site density.

Electrochemical “sense-and-remove” coupling—where the same material simultaneously adsorbs pollutants and generates a quantifiable electrical signal—remains largely underdeveloped. Conducting polymer composites (PANI, PPy, PEDOT) and redox-active MOF-derived electrodes are natural candidates: their ion-exchange and chelation mechanisms inherently alter electrode impedance and voltametric response upon pollutant uptake. Future designs should exploit electrochemical impedance spectroscopy (EIS) and differential pulse voltammetry (DPV) as in operando monitoring tools during adsorption, enabling the real-time quantification of removal efficiency without additional instrumentation.

Photoelectrochemical (PEC) sensing offers a promising pathway for merging photocatalytic degradation with detection: semiconductor composites (TiO_2_, g-C_3_N_4_, BiVO_4_) generate photocurrent changes upon interaction with target pollutants under light irradiation, enabling simultaneous oxidative degradation and quantification. Bio-waste-derived g-C_3_N_4_ analogs from urea or melamine combined with agricultural carbon supports could serve as sustainable PEC platforms for emerging contaminants, including antibiotics and PFASs.

Miniaturized screen-printed electrodes (SPEs) and paper-based electrochemical sensors modified with bio-waste-derived nanocomposites present a clear path to point-of-use water quality monitoring. Integration with smartphones and IoT data platforms would enable the continuous surveillance of contaminated sites, which is relevant for Southeast Asia, Eastern Europe, and regions affected by mining and industrial discharge. Long-term stability under field conditions and validation against reference methods (ICP-MS, AAS) in real water matrices are essential before practical deployment.

Finally, machine learning-assisted signal processing of electrochemical data from multi-electrode arrays could substantially improve selectivity and reduce false positives in complex, multi-contaminant matrices—a challenge that purely materials-based selectivity engineering has not yet fully resolved. Training datasets built from real contaminated water samples (industrial effluents, agricultural runoff, sediment porewater) will be essential for robust model development.

***In summary, the junction of bio-waste products with advanced electrochemical sensing offers a scientifically captivating and economically worthwhile pathway toward next-generation platforms for “sense-and-remove” strategies that are simultaneously sensitive, selective, sustainable, and scalable.***

## 8. Conclusions

The contamination of water and soil by heavy metals poses an escalating risk to ecosystems and human health. Conventional treatment technologies—including precipitation, adsorption, membrane filtration, and biological systems—address either detection or removal, but rarely both simultaneously. In this review, we examine recent progress in advanced and tailored nanocomposites designed for the **integrated “sense-and-remove”** approach to water and soil remediation.

We first survey pollutant categories in water and soil/sediment environments, highlighting their sources, mobility, and toxicological impacts. We then systematically review the materials and methods for **pollutant detection**, encompassing carbon nanomaterials (graphene, GO, rGO, CNTs, carbon dots, GQDs), metal nanoparticles (Au, Ag), metal oxides (TiO_2_, CuO, NiO), MOFs and COFs, conducting polymers (PANI, PPy, PEDOT), and their composites. The detection methods discussed here include electrochemical stripping voltammetry, fluorescence sensing, colorimetry, SERS, photoelectrochemical sensing, and biosensing platforms. Materials and strategies for **pollutant removal** are reviewed in parallel, covering biochar, activated carbon, graphene-based materials, clay minerals, zeolites, polymeric adsorbents, and hybrid electrochemical systems. Particular emphasis is placed on **multifunctional nanocomposites** that integrate both sensing and remediation functions within a single platform—including hydrogel-embedded fluorescent carbon dot systems, stimuli-responsive smart materials, and magnetic composites, enabling easy recovery and regeneration. Key structure–property relationships governing adsorption capacity, sensing selectivity, regeneration efficiency, and operational stability are analyzed.

Despite significant progress in multifunctional sense-and-remove nanocomposites, several challenges continue to hinder their practical implementation. Most of the existing studies have been conducted under controlled laboratory conditions using synthetic solutions, whereas considerably fewer investigations have evaluated performance in real water or soil matrices containing competing ions, natural organic matter, and other interfering species. In addition, issues related to material regeneration, long-term stability, fouling resistance, nanoparticle leaching, economic feasibility, reproducibility, and large-scale production remain insufficiently addressed. Addressing these limitations will be essential to facilitate the translation of multifunctional nanocomposites from proof-of-concept laboratory studies to practical environmental remediation technologies. Future research should therefore focus on developing sustainable, scalable, and environmentally safe materials with improved adsorption capacity, sensing sensitivity, and long-term operational stability.

## Figures and Tables

**Table 1:** Classification and sources of inorganic pollutants, along with their maximum limit values.

Category	Examples	Typical Sources	Maximum Limit	Effects on Human Health/Environment	References
Metals and metalloids	Lead (Pb^2+^)	Industrial effluents, mining, smelting, and motor vehicles	0.01 mg/L	Neurotoxicity; anemia; nephrotoxicity; hypertension	[46,47,48,49,50,51,52,53,54]
	Cadmium (Cd^2+^)		0.003 mg/L	Renal tubular dysfunction; bone demineralization; Group 1 carcinogen; phototoxic	[51]
	Mercury (Hg^2+^)		0.002 mg/L	Neurotoxicity	EPA MCL
	Chromium (Cr^6+^)		0.05 mg/L	Cr^6+^ carcinogenic and mutagenic; dermatitis	[55]
	Arsenic (As^3+^)		0.01 mg/L	Skin lesions; cardiovascular disease; diabetes	[55]
	Zink (Zn^2+^)	Mining; corrosion of plumbing	5 mg/L	Aquatic toxicity; impaired Cu uptake	[56,57]
	Iron (Fe^2+^/Fe^3+^)	Corrosion; acid mine drainage	0.3 mg/L	Hemochromatosis risk	[57]
Inorganic salts (including halides)	Chloride (Cl^−^)	Road salt, geological weathering, and industrial fumes	250 mg/L	-	[58,59,60]
	Fluoride (F^−^)		1.5 mg/L	Dental/skeletal fluorosis at excess	WHO Guideline
	Sulfate (SO_4_^2−^)		250 mg/L	taste	EPA Secondary Standard
Nutrients	Nitrate (NO^3−^)	Agricultural fertilizers, sewage, and animal waste	10 mg/L (as N)	Eutrophication	[61,62,63,64]
	Nitrite (NO^2−^)		1 mg/L (as N)		
Mineral acids and bases	H_2_SO_4_	Food-processing, volcanoes, and acid rain from fossil fuels	pH level 6.5–8.5	Corrosivity; aquatic-life toxicity from pH shift	[65,66]

**Table 2:** Known methods for detecting heavy metals and their advantages and disadvantages.

Method	Main Advantages	Main Limitations
ICP-MS/ICP-OES/AAS/HPLC	Accurate, regulatory validation	Expensive, laboratory-based, sample preparation required
Electrochemical	Portable, sensitive, and low in cost	Electrode fouling, peak overlap, and pH effects
Colorimetric	Simple, visual, field-friendly	Turbidity/color interference and lower precision
Fluorescence	Rapid response and high sensitivity	Background fluorescence, matrix effects, and photobleaching
SERS	Molecular fingerprinting and highly sensitive	Substrate cost, indirect detection often needed, and reproducibility
PEC	Low background, sensitive	Need light source, pH dependence, and photo-corrosion
Biosensing	High selectivity	Biological instability, incubation/amplification steps

**Table 3:** Known materials/methods for detecting heavy metals and their removal mechanism, detection mechanism, and representative performance.

Material Class	Representative Materials	Detection Mechanism	Removal Mechanism	Representative Performance	Refs
Carbon nanomaterials	Graphene, GO, rGO, CNTs, CDs, GQDs, CNFs, biochar-PC	Fast electron transfer at conductive high-area surface + pre-concentration; coordinate M^n+^; fluorescence quenching via photoinduced electron transfer/inner-filter effect	Adsorption via electrostatic attraction, surface complexation with O-groups, π–π stacking, ion exchange	Lignin-PC: Pb^2+^ 250.5/Cd^2+^ 126.4 mg/g (removal); FGO Cd^2+^ LOD 10 nM; N,S-GQD Hg^2+^ LOD 0.14 nM	[111,115,116,117,118,119,120,121,122,123]
noble-metal NPs	Au, Ag	LSPR shift on aggregation (colorimetric); SERS enhancement	Limited standalone capacity—mainly transduction; some catalytic reduction	AuNP Hg^2+^ LOD 10 nM	[124,143,149,150,151]
Metal oxide NPs	TiO_2_, CuO, NiO, Fe_3_O_4_, MnO_2_	Catalytic active sites + interfacial electron transfer; PEC photocurrent change	Surface complexation, ion exchange, magnetic separation (Fe_3_O_4_), photocatalytic ROS degradation of organics	TiO_2_/CPE Hg^2+^ LOD 15.26 nM	[125,126,127,157,158,159,160,161,162,163]
MOFs/COFs	Thiacalixarene-MOF, Bpy-sp^2^c-COF	Luminescent turn-off/on via coordination; electrochemical	Coordination at open metal sites, pore-size selectivity, π–π, electrostatic	MOF Cd^2+^/Pb^2+^ LOD 0.119/0.279 nM; COF Hg^2+^ 415.34 mg/g	[126,127,128,129,164,165,166,167,168,169]
Conducting polymers	PANI, PPy, PEDOT	Redox activity + conductivity/impedance change; ion-imprinting selectivity	Electrostatic, ion exchange, chelation with N/S groups; electrochemically switchable regeneration	IIP-PANI Ni^2+^ LOD 0.00482 µM; PPy Pb^2+^ LOD 0.43 nM	[131,132,133,134,135,170,171,172,173,174,175,176,177,178]
MIPs	Ion-imprinted polymers	Template-shaped cavity recognition coupled to a transducer	Selective rebinding within imprinted cavities	Selective Pb^2+^/Cd^2+^/Hg^2+^/Cu^2+^ recognition	[130,131,132,133,134,179,180,181,182,183,184,185,186,187]
Composites	Carbon/metal-oxide, MOF/polymer, MXene-based	One phase supplies conductivity/signal amplification	Second phase supplies selective binding/adsorption	MWCNT/ZnO Cu/Pb/Hg LOD 0.8/1.2/0.7 ppb; rGO/MXene Zn/Cd/Pb LOD 0.48/0.45/0.29 µg/L	[135,136,137,138,139,140,141,142]

**Table 4:** Comparison of coupled sensing–remediation technologies for pollutant monitoring and removal.

Platform/Technique	Signal Type	Main Functional Materials	Detection Mechanism	Removal Mechanism	Advantages	Limitations	Reference
Fluorescence-based hydrogels	Optical fluorescence	Carbon dots (CDs), quantum dots, MOFs, fluorescent dyes	Fluorescence quenching/enhancement after metal ion binding	Adsorption via chelation, ion exchange, electrostatic attraction	High sensitivity, rapid response, portable visual detection	Fluorescence interference, photobleaching, reduced stability in complex wastewater	[173,175,209,210]
Colorimetric and photonic hydrogels	Color/optical shift	Ion-imprinted polymers, photonic crystals, responsive biopolymers	Visible color or refractive index change upon pollutant binding	Adsorption and physical entrapment	Naked-eye detection, simple operation, low cost	Lower sensitivity compared to fluorescence systems	[171,174,211]
Electrochemical hydrogels	Electrical current/voltage	Graphene, MXenes, conductive polymers, metal nanoparticles	Current generation through anodic stripping voltammetry (ASV) or impedance changes	Electrochemical deposition and adsorption	Ultra-low detection limits, high selectivity, multi-ion analysis	Electrode fouling, expensive instrumentation, signal instability	[189,191,197]
Bio-coupled sensing hydrogels	Biochemical/electrical	Aptamers, enzymes, DNA-functionalized polymers	Conformational or enzymatic changes after target-ion recognition	Selective adsorption and bio-recognition	Exceptional selectivity and specificity	Poor long-term biological stability, enzyme denaturation	[170,212]
MOF-based multifunctional hydrogels	Optical/electrochemical	Luminescent MOFs, hybrid hydrogel networks	Fluorescence or conductivity variation induced by metal binding	Adsorption through porous MOF structures	High surface area, tunable selectivity, multifunctionality	MOF aggregation, water instability, complex synthesis	[172,175]
Magnetic nanocomposite hydrogels	Magnetic/electrochemical	Fe_3_O_4_ nanoparticles, magnetic graphene composites	Signal changes combined with magnetic responsiveness	Magnetic adsorption and easy recovery	Reusability and simple separation	Nanoparticle leakage, lower long-term stability	[31]
Shape-memory and self-reporting hydrogels	Mechanical/optical	Polyacrylic acid, smart polymers, responsive networks	Structural deformation or swelling induced by contaminants	Adsorption through responsive polymer matrices	Real-time visual monitoring and adaptive response	Limited mechanical durability and slow recovery	[174,175]
Photocatalytic detection–degradation systems	Optical/catalytic	TiO_2_, ZnO, MOFs, semiconductor nanocomposites	Pollutant-triggered optical or electrochemical signal	Catalytic degradation via reactive oxygen species (ROS)	Simultaneous sensing and destruction of pollutants	Catalyst deactivation and dependence on light conditions	[163,185,188]
Biopolymer-based multifunctional hydrogels	Optical/electrochemical	Chitosan, alginate, cellulose, gelatin	Fluorescence or electrochemical response after pollutant interaction	Chelation and adsorption via functional groups	Biocompatibility, sustainability, biodegradability	Lower mechanical strength and limited regeneration cycles	[211,212,213]

**Table 5:** Combinations of fluorescent materials and hydrogel matrices used for the simultaneous detection and removal of pollutants.

Material	Target Pollutant	Reference
Carbon dots/cellulose hydrogel	Hg^2+^	[208]
Fluorescent lignin/CNF/carbon dot hydrogel	Cr^6+^	[209]
PVA–carbon nanodot (CND) hydrogel	Fe^3+^	[215]
Chitosan/gelatin fluorescent hydrogel	Cr^6+^	[216]
DNA–chitosan hydrogel	Hg^2+^, Pb^2+^, Cd^2+^, Cu^2+^	[217]

**Table 6:** High-performance adsorption hydrogel materials.

Material	Target Ion	Capacity (mg/g)	Reference
Chitosan/EDTA biochar/acrylic hydrogel (waste cotton)	Pb^2+^/Cu^2+^	1105.78/678.04	[218]
GT-cl-N,N-dimethylacrylamide hydrogel	Hg^2+^/Cr^6+^	666.6/473.9	[219]
Fluorescent lignin/CNF/carbon dot hydrogel	Cr^6+^	599.9	[209]
Diethylaminoethyl dextran hydrogel	Zn^2+^	509.83	[215]
Sodium alginate/PEI composite hydrogel	Cu^2+^/Pb^2+^	322.6/344.8	[216]
Carboxylated chitosan/nanocellulose beads	Pb^2+^	334.92	[217]
Cellulose nanofiber/sodium alginate hydrogel	Pb^2+^	318.47	[220]
PVA/chitosan/polydopamine-GO hydrogel	Cu^2+^/Pb^2+^/Cd^2+^	210.94/236.20/214.98	[221]
Carboxymethyl cellulose-graft-poly(acrylic acid)	Ni^2+^	366.11	[208]
GT/GO composite hydrogel	Pb^2+^/Cd^2+^/Ag^+^	142.50/112.50/132.12	[222]
GT magnetic hydrogel (GT/PVA/GO/Fe_3_O_4_)	Pb^2+^/Cu^2+^	81.78/69.67	[223]
Gelatin–chitosan hydrogel	Hg^2+^	98% removal	[224]

**Table 7:** Representative BET surface areas of conventional and bio-waste-derived materials commonly employed for pollutant adsorption and sensing.

Material	Typical BET Surface Area (m^2^ g^−1^)	Dominant Pore Structure	Reference
Activated carbon	500–2000	Microporous/mesoporous	[228]
Zeolites	300–800	Microporous	[229]
Mesoporous silica	700–1200	Mesoporous	[230]
Biochar	100–1500	Micro-/mesoporous	[231]
Rice husk biochar	200–900	Mesoporous	[232]
Coconut shell activated carbon	800–1800	Microporous	[233]
Chitosan	5–100	Macroporous	[234]
Cellulose aerogels	100–600	Meso-/macroporous	[235]

## Data Availability

No new data were created or analyzed in this study. Data sharing is not applicable to this article.

## Abbreviations

The following abbreviations are used in this manuscript:

AAS	atomic absorption spectroscopy
AOP	advanced oxidation process
ASV	anodic stripping voltammetry
CDI	capacitive deionization
CDs	carbon dots
CNFs	carbon nanofibers
CNMs	carbon nanomaterials
CNTs	carbon nanotubes
COFs	covalent organic frameworks
CPE	carbon paste electrode
CPs	conducting polymers
CQDs	carbon quantum dots
DPV	differential pulse voltammetry
EDTA	ethylenediaminetetraacetic acid
EIS	electrochemical impedance spectroscopy
EPA	US Environmental Protection Agency
GCE	glassy carbon electrode
GO	graphene oxide
GQDs	graphene quantum dots
HPLC	high-performance liquid chromatography
ICP-MS	inductively coupled plasma mass spectrometry
ICP-OES	inductively coupled plasma optical emission spectroscopy
IIPANI	ion imprinted polyaniline
LCA	life cycle assessment
LMOF	luminescent metal-organic framework
LSASV	linear sweep anodic stripping voltammetry
MIPs	molecularly imprinted polymers
MOFs	metal-organic frameworks
MOs	metal oxide nanoparticles
MWCNTs	multi-walled carbon nanotubes
NC	nitrogen-doped carbon
NPs	nanoparticles
PA	phytic acid
PAHs	polycyclic aromatic hydrocarbons
PANI	polyaniline
PASP	polyaspartic acid
PC	porous carbon
PCI	photoelectron chemical ionization
PEC	photoelectrochemical
PEDOT	poly(3,4-ethylenedioxythiophene)
PET	photo-induced electron transfer
PFAS	per- and polyfluoroalkyl substances
PI	photon ionization
PPy	polypyrrole
PSS	poly(styrenesulfonate)
rGO	reduced graphene oxide
ROS	reactive oxygen species
SERS	surface-enhanced Raman scattering
SESR	surfactant-enhanced soil remediation
SPR	surface plasmon resonance
SWV	square-wave voltammetry
VUP-TOF-MS	vacuum ultraviolet photoionization time-of-flight mass spectrometry
WHO	World Health Organization
SEM	scanning electron microscopy

## References

[1] Mishra R.K. (2023). Fresh water availability and its global challenge. Br. J. Multidiscip. Adv. Stud..

[2] Anastasi C. (2023). Desalination and Water Security.

[3] Singh V. (2024). Water Resources.

[4] Saravanan A., Senthil Kumar P., Jeevanantham S., Karishma S., Tajsabreen B., Yaashikaa P., Reshma B. (2021). Effective water/wastewater treatment methodologies for toxic pollutants removal: Processes and applications towards sustainable development. Chemosphere.

[5] Zhao J., Wang Y., Zhang X., Liu Q. (2022). Industrial and agricultural water use efficiency and influencing factors in the process of urbanization in the middle and lower reaches of the yellow river basin, china. Land.

[6] Lu F., Astruc D. (2020). Nanocatalysts and other nanomaterials for water remediation from organic pollutants. Coord. Chem. Rev..

[7] Wang J., Wang Z., Vieira C.L., Wolfson J.M., Pingtian G., Huang S. (2019). Review on the treatment of organic pollutants in water by ultrasonic technology. Ultrason. Sonochemistry.

[8] Ahn C., Connor R., UNESCO World Water Assessment Programme (2025). The United Nations World Water Development Report 2025: Mountains and Glaciers—Water Towers.

[9] Javan K., Altaee A., BaniHashemi S., Darestani M., Zhou J., Pignatta G. (2024). A review of interconnected challenges in the water–energy–food nexus: Urban pollution perspective towards sustainable development. Sci. Total Environ..

[10] Peters N.E., Meybeck M. (2000). Water quality degradation effects on freshwater availability: Impacts of human activities. Water Int..

[11] World Health Organization (2019). Drinking-Water.

[12] Sarker A., Masud M.A.A., Deepo D.M., Das K., Nandi R., Ansary M.W.R., Islam A.R.M.T., Islam T. (2023). Biological and green remediation of heavy metal contaminated water and soils: A state-of-the-art review. Chemosphere.

[13] Batool M., Zafar M.N. (2025). Synthesis of a bisbs 3 @bisbo 4 /CNH nanocomposite for wastewater treatment and electrochemical application. Mater. Adv..

[14] Zhang Y., Yang J., Chen J., Luo H., Ma H., Pu S. (2025). Enhancement of 2,4,6-trichlorophenol degradation in groundwater via natural pyrite-activated percarbonate: Mechanistic insights and efficiency evaluation. J. Environ. Chem. Eng..

[15] Kumar S., Mehta P. (2023). Ultralow-loss Optical Waveguides through Balancing Deep-Blue TADF and Orange Room Temperature Phosphorescence in Hybrid Antimony Halide Microstructures. Angew. Chem. Int. Ed..

[16] Saleh T.A., Mustaqeem M., Khaled M. (2022). Water treatment technologies in removing heavy metal ions from wastewater: A review. Environ. Nanotechnol. Monit. Manag..

[17] Samanta P., Desai A.V., Let S., Ghosh S.K. (2019). Advanced porous materials for sensing, capture and detoxification of organic pollutants toward water remediation. ACS Sustain. Chem. Eng..

[18] Kumar M., Sridharan S., Sawarkar A.D., Shakeel A., Anerao P., Mannina G., Sharma P., Pandey A. (2023). Current research trends on emerging contaminants pharmaceutical and personal care products (ppcps): A comprehensive review. Sci. Total Environ..

[19] Yuan Z., Nag R., Cummins E. (2022). Human health concerns regarding microplastics in the aquatic environment-from marine to food systems. Sci. Total Environ..

[20] Crini G., Lichtfouse E. (2019). Advantages and disadvantages of techniques used for wastewater treatment. Environ. Chem. Lett..

[21] Mittal H., Kumar V., Saruchi, Ray S.S. (2016). Adsorption of methyl violet from aqueous solution using gum xanthan/Fe_3_O_4_ based nanocomposite hydrogel. Int. J. Biol. Macromol..

[22] Poerio T., Lavorato C., Severino A., Russo B., Molinari R., Argurio P., Figoli A. (2024). Combined membrane separation and photocatalysis process for the recovery and decomposition of micro/nanoplastics from polyester fabrics. J. Environ. Chem. Eng..

[23] Nthunya L.N., Mbakop S., Mhlanga S.D. (2021). Emerging nanoenhanced membrane-based hybrid processes for complex industrial wastewater treatment. Membrane-Based Hybrid Processes for Wastewater Treatment.

[24] Adeoye J.B., Tan Y.H., Lau S.Y., Tan Y.Y., Chiong T., Mubarak N.M., Khalid M. (2024). Advanced oxidation and biological integrated processes for pharmaceutical wastewater treatment: A review. J. Environ. Manag..

[25] Long F., Ghani D., Huang R., Zhao C. (2025). Versatile electrode materials applied in the electrochemical advanced oxidation processes for wastewater treatment: A systematic review. Sep. Purif. Technol..

[26] Babu Ponnusami A., Sinha S., Ashokan H., V Paul M., Hariharan S.P., Arun J., Gopinath K., Hoang Le Q., Pugazhendhi A. (2023). Advanced oxidation process (AOP) combined biological process for wastewater treatment: A review on advancements, feasibility and practicability of combined techniques. Environ. Res..

[27] Elmahdi A. (2024). Addressing water scarcity in agricultural irrigation: By exploring alternative water resources for sustainable irrigated agriculture. Irrig. Drain..

[28] Agrawal R., Agrawal S., Samadhiya A., Kumar A., Luthra S., Jain V. (2024). Adoption of green finance and green innovation for achieving circularity: An exploratory review and future directions. Geosci. Front..

[29] Akhtar M.S., Ali S., Zaman W. (2024). Innovative Adsorbents for Pollutant Removal: Exploring the Latest Research and Applications. Molecules.

[30] Mohammadzadeh Kakhki R. (2026). Introduction to Advanced Materials for Environ-mental Remediation. Nanomaterials for Environmental Remediation.

[31] Tiwari M., Tripathy D.B. (2023). Soil Contaminants and Their Removal through Surfactant-Enhanced Soil Remediation: A Comprehensive Review. Sustainability.

[32] Faysal Hossain M., Akther N., Zhou Y. (2020). Recent advancements in graphene adsorbents for wastewater treatment: Current status and challenges. Chin. Chem. Lett..

[33] Younas F., Mustafa A., Farooqi Z.U.R., Wang X., Younas S., Mohy-Ud-Din W., Ashir Hameed M., Mohsin Abrar M., Maitlo A.A., Noreen S. (2021). Current and emerging adsorbent technologies for wastewater treatment: Trends, limitations, and environmental implications. Water.

[34] Irannajad M., Kamran Haghighi H. (2021). Removal of heavy metals from polluted solutions by zeolitic adsorbents: A review. Environ. Process..

[35] Xu Y., Fan Z., Li X., Yang S., Wang S., Wang J., Zheng A., Shu R. (2024). Cooperative production of monophenolic chemicals and carbon adsorption materials from cascade pyrolysis of acid hydrolysis lignin. Bioresour. Technol..

[36] Kayan G.Ö., Kayan A. (2021). Composite of natural polymers and their adsorbent properties on the dyes and heavy metal ions. J. Polym. Environ..

[37] Spoială A., Ilie C.I., Ficai D., Ficai A., Andronescu E. (2021). Chitosan-based nanocomposite polymeric membranes for water purification—A review. Materials.

[38] Laishram D., Kim S., Lee S., Park S. (2025). Advancements in biochar as a sustainable adsorbent for water pollution mitigation. Adv. Sci..

[39] Alsawy T., Rashad E., El-Qelish M., Mohammed R.H. (2022). A comprehensive review on the chemical regeneration of biochar adsorbent for sustainable wastewater treatment. npj Clean Water.

[40] Bobde P.V., Sharma A.K., Kumar R., Pandey J.K., Wadhwa S. (2023). Recent advances on sustainable removal of emerging contaminants from water by bio-based adsorbents. N. J. Chem..

[41] Gadore V., Ahmaruzzaman M. (2021). Smart materials for remediation of aqueous environmental contaminants. J. Environ. Chem. Eng..

[42] Wang Y., Cheng Y., Liu H., Guo Q., Dai C., Zhao M., Liu D. (2023). A review on applications of artificial intelligence in wastewater treatment. Sustainability.

[43] Ramakrishnan T., Kumar S.S., Chelladurai S.J.S., Gnanasekaran S., Sivananthan S., Geetha N.K., Arthanari R., Assefa G.B. (2022). Recent developments in stimuli responsive smart materials and applications: An overview. J. Nanomater..

[44] Gahrouei A.E., Rezapour A., Pirooz M., Pourebrahimi S. (2024). From classic to cutting-edge solutions: A comprehensive review of materials and methods for heavy metal removal from water environments. Desalin. Water Treat..

[45] Srivastav A.L., Ranjan M. (2020). Chapter 1-Inorganic water pollutants. Inorganic Pollutants in Water.

[46] Yu D. (2025). Lead exposure in the 21st century: Modeling a path from crisis to prevention. Eco-Environ. Health.

[47] World Health Organization Mercury and Health. WHO Fact Sheet. https://www.who.int/news-room/fact-sheets/detail/mercury-and-health.

[48] Kennedy J., Calikanzaros E., Landrigan P.J., Badot P.-M., Cinar M., Safa A., Schomaker R.M., Lloret J., Raps H., Racault M.-F. (2025). Methylmercury contamination in Mediterranean seafood: Exposure assessment and cost of illness implications. Sci. Total Environ..

[49] Hutton M. (1983). Sources of cadmium in the environment. Ecotoxicol. Environ. Saf..

[50] Chețan F., Moraru P.I., Rusu T., Șimon A., Dinca L., Murariu G. (2025). From contamination to mitigation: Addressing cadmium pollution in agricultural soils. Agriculture.

[51] UK Health Security Agency Cadmium: Properties, Incident Management and Toxicology. https://www.gov.uk/government/publications/cadmium-properties-incident-management-and-toxicology/cadmium-general-information.

[52] Bao S., Yang W., Wang Y., Yu Y., Sun Y. (2020). One-pot synthesis of magnetic graphene oxide composites as an efficient and recoverable adsorbent for Cd(II) and Pb(II) removal from aqueous solution. J. Hazard. Mater..

[53] Mladin G., Ciopec M., Negrea A., Duteanu N., Negrea P., Ianasi P., Ianași C. (2022). Silica-Iron Oxide Nanocomposite Enhanced with Porogen Agent Used for Arsenic Removal. Materials.

[54] Fan L., Luo C., Sun M., Li X., Qiu H. (2013). Highly selective adsorption of lead ions by water-dispersible magnetic chitosan/graphene oxide composites. Colloids Surf. B Biointerfaces.

[55] World Health Organization Arsenic. WHO Fact Sheet. https://www.who.int/news-room/fact-sheets/detail/arsenic.

[56] Petruzzelli G., Pezzarossa B., Pedron F. (2025). The fate of chemical contaminants in soil with a view to potential risk to human health: A Review. Environments.

[57] Xie Y., Cheng D., Liu X., Han A. (2019). Green hydrothermal synthesis of N-doped carbon dots from biomass highland barley for the detection of Hg^2+^. Sensors.

[58] Wang Y., Liu X., Wang L., Li H., Zhang S., Yang J., Liu N., Han X. (2023). Effects of Long-Term Application of Cl-Containing Fertilizers on Chloride Content and Acidification in Brown Soil. Sustainability.

[59] Eletta O.A.A., Ajayi O.A., Ogunleye O.O., Akpan I.C. (2016). Adsorption of cyanide from aqueous solution using calcinated eggshells: Equilibrium and optimisation studies. J. Environ. Chem. Eng..

[60] Lin K.-Y.A., Liu Y.-T., Chen S.-Y. (2016). Adsorption of fluoride to UiO-66-NH2 in water: Stability, kinetic, isotherm and thermodynamic studies. J. Colloid Interface Sci..

[61] Xia S., Liang S., Qin Y., Chen W., Xue B., Zhang B., Xu G. (2023). Significant Improvement of Adsorption for Phosphate Removal by Lanthanum-Loaded Biochar. ACS Omega.

[62] Battas A., El Gaidoumi A., Ksakas A., Kherbeche A. (2019). Adsorption Study for the Removal of Nitrate from Water Using Local Clay. Sci. World J..

[63] Liu Y., Zhang X., Wang J. (2022). A critical review of various adsorbents for selective removal of nitrate from water: Structure, performance and mechanism. Chemosphere.

[64] Zhang X., Liu X., Zhang Z., Chen Z. (2021). Removal of phosphate from aqueous solution by chitosan coated and lanthanum loaded biochar derived from urban dewatered sewage sludge. Water Sci. Technol..

[65] Tolonen E.-T., Hu T., Rämö J., Lassi U. (2016). The removal of sulphate from mine water by precipitation as ettringite and the utilisation of the precipitate as a sorbent for arsenate removal. J. Environ. Manag..

[66] Dou W., Zhou Z., Jiang L.-M., Jiang A., Huang R., Tian X., Zhang W., Chen D. (2017). Sulfate removal from wastewater using ettringite precipitation: Magnesium ion inhibition and process optimization. J. Environ. Manag..

[67] Niu A., Lin C. (2021). Managing soils of environmental significance: A critical review. J. Hazard. Mater..

[68] Correia A.A.S., Rasteiro M.G. (2025). A Review of persistent soil contaminants: Assessment and remediation strategies. Environments.

[69] Zhu Y.G., Shaw G. (2000). Soil contamination with radionuclides and potential remediation. Chemosphere.

[70] De Silva S., Carson P., Indrapala D.V., Warwick B., Reichman S.M. (2023). Land application of industrial wastes: Impacts on soil quality, biota, and human health. Environ. Sci. Pollut. Res..

[71] Nuruzzaman, Bahar M., Naidu R. (2025). Diffuse soil pollution from agriculture: Impacts and remediation. Sci. Total Environ..

[72] Dehkordi M.M., Nodeh Z.P., Dehkordi K.S., Salmanvandi H., Khorjestan R.R., Ghaffarzadeh M. (2024). Soil, air, and water pollution from mining and industrial activities: Sources of pollution, environmental impacts, and prevention and control methods. Results Eng..

[73] World Health Organization Solid Waste. WHO Compendium on Health and Environment. https://www.who.int/tools/compendium-on-health-and-environment/solid-waste.

[74] Hamilton S.F., Sproul T.W., Sunding D., Zilberman D. (2013). Environmental policy with collective waste disposal. J. Environ. Econ. Manag..

[75] Strawn D.G. (2021). Sorption mechanisms of chemicals in soils. Soil Syst..

[76] Kleber M., Bourg I.C., Coward E.K., Hansel C.M., Myneni S.C.B., Nunan N. (2021). Dynamic interactions at the mineral–organic matter interface. Nat. Rev. Earth Environ..

[77] Li Q., Wang Y., Li Y., Li L., Tang M., Hu W., Chen L., Ai S. (2022). Speciation of heavy metals in soils and their immobilization at micro-scale interfaces among diverse soil components. Sci. Total Environ..

[78] National Academies of Sciences, Engineering, and Medicine (2024). Exploring Linkages Between Soil Health and Human Health.

[79] Jiang W., Sheng Y. (2025). Soil–groundwater environmental quality and water resource: Assessment of contaminant sources, species, and transformations. Water.

[80] Wu X., Fan J., Sun L., Zhang H., Xu Y., Yao Y., Yan X., Zhou J., Jia Y., Chi W. (2021). Wind erosion and its ecological effects on soil in the northern piedmont of the Yinshan Mountains. Ecol. Indic..

[81] Yang M., Tian X., Guo Z., Chang C., Li J., Guo Z., Li H., Liu R., Wang R., Li Q. (2023). Wind erosion induced low-density microplastics migration at landscape scale in a semi-arid region of northern China. Sci. Total Environ..

[82] Kourtchev I., McGillen M.R., Wenger J., Donahue N.M. (2025). Rethinking environmental boundaries for contaminants of emerging concern. Atmos. Environ..

[83] Montero-Montoya R., López-Vargas R., Arellano-Aguilar O. (2018). Volatile organic compounds in air: Sources, distribution, exposure and associated illnesses in children. Ann. Glob. Health.

[84] Fang S., Hua C., Yang J., Liu F., Wang L., Wu D., Ren L. (2025). Combined pollution of soil by heavy metals, microplastics, and pesticides: Mechanisms and anthropogenic drivers. J. Hazard. Mater..

[85] Briffa J., Sinagra E., Blundell R. (2020). Heavy metal pollution in the environment and their toxicological effects on humans. Heliyon.

[86] Dong W., Zhang Y., Quan X. (2020). Health risk assessment of heavy metals and pesticides: A case study in the main drinking water source in Dalian, China. Chemosphere.

[87] Gimeno-García E., Andreu V., Boluda R. (1996). Heavy metals incidence in the application of inorganic fertilizers and pesticides to rice farming soils. Environ. Pollut..

[88] Rey-Martínez N., Guisasola A., Baeza J.A. (2022). Assessment of the significance of heavy metals, pesticides and other contaminants in recovered products from water resource recovery facilities. Resour. Conserv. Recycl..

[89] Pulze S., Presti N., Anastasio A. (2025). Impact of volcanic eruptions on heavy metal contamination in the food chain. Ital. J. Food Saf..

[90] Mustapha L.S., Obayomi O.V., Obayomi K.S. (2026). A comprehensive review on potential heavy metals in the environment: Persistence, bioaccumulation, ecotoxicology, and agricultural impacts. Ecol. Front..

[91] Lang T., Lipp A.-M., Wechselberger C. (2025). Xenobiotic toxicants and particulate matter: Effects, mechanisms, impacts on human health, and mitigation strategies. J. Xenobiot..

[92] Shekhar C., Khosya R., Sharma A.K., Thakur K., Mahajan D., Kumar R., Kumar S., Sharma A.K. (2025). A systematic review on health risks of water pollutants: Classification, effects and innovative solutions for conservation. Toxicol. Res..

[93] Jochim M.A., Dickens R.S. (2002). Soils, sediments, and geomorphology. The Archaeologist’s Laboratory. Interdisciplinary Contributions to Archaeology.

[94] Hossen M.A., Mostafa M.G. (2026). Emerging pollutants and heavy metals in sediments: An overview. Cont. Shelf Res..

[95] Eggleton J., Thomas K.V. (2004). A review of factors affecting the release and bioavailability of contaminants during sediment disturbance events. Environ. Int..

[96] Chiaia-Hernández A.C., Casado-Martinez C., Lara-Martin P., Bucheli T.D. (2022). Sediments: Sink, archive, and source of contaminants. Environ. Sci. Pollut. Res. Int..

[97] Zoumis T., Schmidt A., Grigorova L., Calmano W. (2001). Contaminants in sediments: Remobilisation and demobilisation. Sci. Total Environ..

[98] Kalnejais L.H., Martin W.R., Signell R.P., Bothner M. (2007). Role of sediment resuspension in the remobilization of particulate-phase metals from coastal sediments. Environ. Sci. Technol..

[99] Dang D.H., Layglon N., Ferretto N., Omanović D., Mullot J.-U., Lenoble V., Mounier S., Garnier C. (2020). Kinetic processes of copper and lead remobilization during sediment resuspension of marine polluted sediments. Sci. Total Environ..

[100] Zhang Y., Labianca C., Chen L., De Gisi S., Notarnicola M., Guo B., Sun J., Ding S., Wang L. (2021). Sustainable ex-situ remediation of contaminated sediment: A review. Environ. Pollut..

[101] Taylor K.G., Owens P.N. (2009). Sediments in urban river basins: A review of sediment-contaminant dynamics in an environmental system conditioned by human activities. J. Soils Sediments.

[102] Kumar M., Jain V., Yamanaka T., Li Y., Bhattacharya P. (2018). Contaminant transport and fate in freshwater systems–Integrating the fields of geochemistry, geomorphology and nanotechnology. Groundw. Sustain. Dev..

[103] Xu Q., Wu B., Chai X. (2022). In situ remediation technology for heavy metal contaminated sediment: A review. Int. J. Environ. Res. Public Health.

[104] Owens P.N., Batalla R.J., Collins A.J., Gomez B., Hicks D.M., Horowitz A.J., Kondolf G.M., Marden M., Page M.J., Peacock D.H. (2005). Fine-grained sediment in river systems: Environmental significance and management issues. River Res. Appl..

[105] Biswas B., Qi F., Biswas J.K., Wijayawardena A., Khan M.A.I., Naidu R. (2018). The fate of chemical pollutants with soil properties and processes in the climate change paradigm—A review. Soil Syst..

[106] Chang J., Zhou G., Christensen E.R., Heideman R., Chen J. (2014). Graphene-based sensors for detection of heavy metals in water: A review. Anal. Bioanal. Chem..

[107] Moo J.G.S., Khezri B., Webster R.D., Pumera M. (2014). Graphene Oxides Prepared by Hummers’, Hofmann’s, and Staudenmaier’s Methods: Dramatic Influences on Heavy-Metal-Ion Adsorption. ChemPhysChem.

[108] Xuan X., Hossain M.F., Park J.Y. (2016). A fully integrated and miniaturized heavy-metal-detection sensor based on micro-patterned reduced graphene oxide. Sci. Rep..

[109] Sitko R., Zawisza B., Malicka E. (2012). Modification of carbon nanotubes for preconcentration, separation and determination of trace-metal ions. TrAC Trends Anal. Chem..

[110] Gontrani L., Pulci O., Carbone M., Pizzoferrato R., Prosposito P. (2021). Detection of heavy metals in water using graphene oxide quantum dots: An experimental and theoretical study. Molecules.

[111] Zhao D., Wang T., Han D., Rusinek C., Steckl A.J., Heineman W.R. (2015). Electrospun carbon nanofiber modified electrodes for stripping voltammetry. Anal. Chem..

[112] Wang A., Zheng Z., Li R., Hu D., Lu Y., Luo H., Yan K. (2019). Biomass-derived porous carbon highly efficient for removal of Pb(II) and Cd(II). Green Energy Environ..

[113] Jaswal N., Kour J., Kumar P. (2026). Biomass-Derived Activated Carbon as a Fluorescent Probe for Sensitive Detection of Mercury (II) in Wastewater. J. Fluoresc..

[114] Thiruppathi A.R., Sidhureddy B., Keeler W., Chen A. (2017). Facile one-pot synthesis of fluorinated graphene oxide for electrochemical sensing of heavy metal ions. Electrochem. Commun..

[115] Tan Q., Li X., Wang L., Zhao J., Yang Q., Sun P., Deng Y., Shen G. (2022). One-step synthesis of highly fluorescent carbon dots as fluorescence sensors for the parallel detection of cadmium and mercury ions. Front. Chem..

[116] George H.S., Selvaraj H., Ilangovan A., Chandrasekaran K., Kannan V.R., Parthipan P., Almutairi B.O., Balu R. (2023). Green synthesis of biomass derived carbon dots via microwave-assisted method for selective detection of Fe^3+^ ions in an aqueous medium. Inorg. Chem. Commun..

[117] Anh N.T.N., Chowdhury A.D., Doong R.A. (2017). Highly sensitive and selective detection of mercury ions using N, S-codoped graphene quantum dots and its paper strip-based sensing application in wastewater. Sens. Actuators B Chem..

[118] Bressi V., Celesti C., Ferlazzo A., Len T., Moulaee K., Neri G., Luque R., Espro C. (2024). Waste-derived carbon nanodots for fluorimetric and simultaneous electrochemical detection of heavy metals in water. Environ. Sci. Nano.

[119] Guo X., Yun Y., Shanov V.N., Halsall H.B., Heineman W.R. (2011). Determination of trace metals by anodic stripping voltammetry using a carbon nanotube tower electrode. Electroanalysis.

[120] Kumar N., Tran H., Shanov V.N., Alvarez N.T. (2024). Electrochemical detection of lead ions using carbon nanotube post electrodes. Chem. Eng. J..

[121] Zhao D., Siebold D., Alvarez N.T., Shanov V.N., Heineman W.R. (2017). Carbon nanotube thread electrochemical cell: Detection of heavy metals. Anal. Chem..

[122] Robinson J.E., Heineman W.R., Sagle L.B., Meyyappan M., Koehne J.E. (2016). Carbon nanofiber electrode array for the detection of lead. Electrochem. Commun..

[123] Morris A., Serrano N., Díaz-Cruz J.M., Bendavid A., Chen M., Vepsäläinen M. (2021). Vibrating boron-doped diamond electrode: A new, durable and highly sensitive tool for the detection of cadmium. Anal. Chim. Acta.

[124] Kim S. (2023). Development of boron doped diamond electrodes material for heavy metal ion sensor with high sensitivity and durability. Case Stud. Chem. Environ. Eng..

[125] Wu L., Liu X., Yu X., Xu S., Zhang S., Guo S. (2022). Fabrication of boron-doped diamond film electrode for detecting trace lead content in drinking water. Materials.

[126] Tüzün E., Atun G. (2023). Individual and simultaneous determination of heavy metal ions using carbon paste electrode modified with titania nanoparticles. Electrocatalysis.

[127] Tamilselvan S., Soniya R.M., Vasantharaja R., Kannan M., Supriya S., Batvari B.P.D., Ramesh T., Govindaraju K. (2022). Silver nanoparticles based spectroscopic sensing of eight metal ions in aqueous solutions. Environ. Res..

[128] Ali D.S.B., Krid F., Nacef M., Boussaha E.H., Chelaghmia M.L., Tabet H., Selaimia R., Atamnia A., Affoune A.M. (2023). Green synthesis of copper oxide nanoparticles using Ficus elastica extract for the electrochemical simultaneous detection of Cd^2+^, Pb^2+^, and Hg^2+^. RSC Adv..

[129] Ma L., Pei W.Y., Yang J., Ma J.F. (2024). A new thiacalix[4]arene-based metal-organic framework as an efficient electrochemical sensor for trace detection of Cd^2+^ and Pb^2+^. Food Chem..

[130] Zhu B., Zhu L., Deng S., Wan Y., Qin F., Han H., Luo J. (2023). A fully π-conjugated covalent organic framework with dual binding sites for ultrasensitive detection and removal of divalent heavy metal ions. J. Hazard. Mater..

[131] Setiyanto H., Purwaningsih D.R., Saraswaty V., Mufti N., Zulfikar M.A. (2022). Highly selective electrochemical sensing based on electropolymerized ion imprinted polyaniline (IIPANI) on a bismuth modified carbon paste electrode (CPE-Bi) for monitoring Nickel(II) in river water. RSC Adv..

[132] Velempini T., Pillay K., Mbianda X.Y., Arotiba O.A. (2018). Application of a polypyrrole/carboxy methyl cellulose ion imprinted polymer in the electrochemical detection of mercury in water. Electroanalysis.

[133] Zhang H., Li Y., Zhang Y., Wu J., Li S., Li L. (2023). A Disposable Electrochemical Sensor for Lead Ion Detection Based on In Situ Polymerization of Conductive Polypyrrole Coating. J. Electron. Mater..

[134] Anandhakumar S., Mathiyarasu J., Phani K.L.N., Yegnaraman V. (2011). Simultaneous determination of cadmium and lead using PEDOT/PSS modified glassy carbon electrode. Am. J. Anal. Chem..

[135] Alshawi J.M., Mohammed M.Q., Alesary H.F., Ismail H.K., Barton S. (2022). Voltammetric determination of Hg^2+^, Zn^2+^, and Pb^2+^ ions using a PEDOT/NTA-modified electrode. ACS Omega.

[136] Zhou X. (2024). Electrochemical detection of heavy metal ions in water using MWCNT/ZnO nanocomposite. Int. J. Electrochem. Sci..

[137] Verma M., Kumari A., Bahuguna G., Singh V., Pareek V., Dhamija A., Shukla S., Ghosh D., Gupta R. (2024). SnO2–MWCNT and SnO2–rGO nanocomposites for selective electrochemical detection in a mixture of heavy metal ions. ACS Appl. Nano Mater..

[138] Liu Y., Wu S., Xiong W., Li H. (2023). Interface Co-assembly synthesis of magnetic Fe_3_O_4_@mesoporous carbon for efficient electrochemical detection of Hg(II) and Pb(II). Adv. Mater. Interfaces.

[139] Bhuvaneswari K., Radha S., Sreeja B.S., Kumar P.S. (2023). Development of in-situ electrochemical heavy metal ion sensor using integrated 1D/0D/1D hybrid by MWCNT and CQDs supported MnO2 nanomaterial. Environ. Res..

[140] Milikić J., Savić M., Janošević Ležaić A., Šljukić B., Ćirić-Marjanović G. (2024). Electrochemical sensing of cadmium and lead ions in water by MOF-5/PANI composites. Polymers.

[141] Zhao J., Long Y., He C., Yang H., Zhao S., Luo X., Huo D., Hou C. (2023). Simultaneous electrochemical detection of Cd^2+^ and Pb^2+^ based on an MOF-derived carbon composite linked with multiwalled carbon nanotubes. ACS Sustain. Chem. Eng..

[142] Dai H., Wang N., Wang D., Ma H., Lin M. (2016). An electrochemical sensor based on phytic acid functionalized polypyrrole/graphene oxide nanocomposites for simultaneous determination of Cd(II) and Pb(II). Chem. Eng. J..

[143] Chen Y., Zhao P., Liang Y., Ma Y., Liu Y., Zhao J., Hou J., Hou C., Huo D. (2023). A sensitive electrochemical sensor based on 3D porous melamine-doped rGO/MXene composite aerogel for the detection of heavy metal ions in the environment. Talanta.

[144] U.S. EPA Method 6020B. Inductively Coupled Plasma–Mass Spectrometry. https://www.epa.gov/sites/default/files/2015-12/documents/6020b.pdf.

[145] U.S. EPA Method 245.1. Determination of Mercury in Water by Cold Vapor Atomic Absorption Spectrometry. https://www.epa.gov/sites/default/files/2015-06/documents/epa-245.1.pdf.

[146] Shao X., Yang D., Wang M., Yue Q. (2023). A colorimetric detection of Hg^2+^ based on gold nanoparticles synthesized oxidized N-methylpyrrolidone as a reducing agent. Sci. Rep..

[147] Bu L., Peng J., Peng H., Liu S., Xiao H., Liu D., Pan Z., Chen Y., Chen F., He Y. (2016). Fluorescent carbon dots for the sensitive detection of Cr(VI) in aqueous media and their application in test papers. RSC Adv..

[148] Xing S., Zheng K., Shi L., Kang K., Peng Z., Zhang X., Liu B., Yang H., Yue G. (2024). Fluorescence detection of Pb^2+^ in environmental water using biomass carbon quantum dots modified with acetamide-glycolic acid deep eutectic solvent. Molecules.

[149] Long X., Li R., Xiang J., Wu S., Wang J. (2022). Ultrabright carbon dots as a fluorescent nano sensor for Pb^2+^ detection. RSC Adv..

[150] Tian C., Zhao L., Zhu J., Zhang S. (2021). Ultrasensitive detection of trace Hg^2+^ by SERS aptasensor based on dual recycling amplification in water environment. J. Hazard. Mater..

[151] Liu S., Zhan J., Cai B. (2024). Recent advances in photoelectrochemical platforms based on porous materials for environmental pollutant detection. RSC Adv..

[152] Li S., Xu J., Li H. (2024). Highly sensitive detection of Pb^2+^ in the environment with DNAzyme and rolling circle amplification reaction. Spectrochim. Acta A.

[153] Memon A.G., Zhou X., Xing Y., Wang R., Liu L., Khan M., He M. (2019). Label-free colorimetric nanosensor with improved sensitivity for Pb^2+^ in water by using a truncated 8-17 DNAzyme. Front. Environ. Sci. Eng..

[154] Cai B., Ren T., Yu X., Lv W., Liang Y. (2024). Aptamer-functionalized gold nanoparticles for mercury ion detection in a colorimetric assay based on color change time as signal readout. Microchim. Acta.

[155] Huggins T.M., Haeger A., Biffinger J.C., Ren Z.J. (2016). Granular biochar compared with activated carbon for wastewater treatment and resource recovery. Water Res..

[156] Zhang Y., Cao B., Zhao L., Sun L., Gao Y., Li J. (2018). Biochar-supported reduced graphene oxide composite for adsorption and coadsorption of atrazine and lead ions. Appl. Surf. Sci..

[157] Meng F., Wang Y., Wei Y. (2025). Advancements in Biochar for Soil Remediation of Heavy Metals and/or Organic Pollutants. Materials.

[158] Iqbal M.S., Aslam A.A., Iftikhar R., Junaid M., Imran S.M., Nazir M.S. (2023). The potential of functionalized graphene-based composites for removing heavy metals and organic pollutants. J. Water Process Eng..

[159] Alfei S., Zuccari G. (2025). Carbon-Nanotube-Based Nanocomposites in Environmental Remediation: An Overview of Typologies and Applications and an Analysis of Their Paradoxical Double-Sided Effects. J. Xenobiot..

[160] Tamura H., Katayama N., Furuichi R. (1996). Modeling of Ion-Exchange Reactions on Metal Oxides with the Frumkin Isotherm. 1. Acid−Base and Charge Characteristics of MnO_2_, TiO_2_, Fe_3_O_4_, and Al_2_O_3_ Surfaces and Adsorption Affinity of Alkali Metal Ions. Environ. Sci. Technol..

[161] Al Miad A., Saikat S.P., Alam K., Sahadat H., Bahadur N.M., Ahmed S. (2024). Metal oxide-based photocatalysts for the efficient degradation of organic pollutants for a sustainable environment: A review. Nanoscale Adv..

[162] Kamel A.H., Abd-Rabboh H.S.M., El-Fattah A.A., Boudghene S.G., Adeida L. (2025). Metal oxides and their composites for the remediation of organic pesticides: Advanced photocatalytic and adsorptive solutions. RSC Adv..

[163] Qumar U., Hassan J.Z., Bhatti R.A., Raza A., Nazir G., Nabgan W. (2022). Photocatalysis vs adsorption by metal oxide nanoparticles. J. Mater. Sci. Technol..

[164] Khoo P.S., Ilyas R.A., Aiman A., Wei J.S., Yousef A., Anis N., Zuhri M.Y.M., Abral H., Sari N.H., Syafri E. (2024). Revolutionizing wastewater treatment: A review on the role of advanced functional bio-based hydrogels. Int. J. Biol. Macromol..

[165] Yan Z., Cai Y., Zhang J., Zhao Y. (2022). Fluorescent sensor arrays for metal ions detection: A review. Measurement.

[166] Liu D.-M., Dong C., Jiang H.-L. (2025). Nanomaterial-based sensors for heavy metal ions analysis. Microchem. J..

[167] Lu S., Yang B. (2022). Carbon dots are shining in the world. SmartMat.

[168] Batool M., Junaid H.M., Tabassum S., Kanwal F., Abid K., Fatima Z., Shah A.T. (2020). Metal Ion Detection by Carbon Dots—A Review. Crit. Rev. Anal. Chem..

[169] Fan J., Kang L., Liu D., Zhang S. (2023). Modification of Carbon Dots for Metal-Ions Detection. ChemistrySelect.

[170] Theyagarajan K. (2026). MXene- and MOF-Based Hydrogels: Emerging Platforms for Electrochemical Biosensing and Health Monitoring. Biosensors.

[171] Liu Z., Zhang M., Yang H., Zhang C., Hou Y., Wang J., Fei P., Feng F., Feng Y. (2026). Recent Advances in Bio-Based Fluorescent Hydrogels for Adsorption and Sensing of Toxic Heavy Metal Ions. Molecules.

[172] Zhou X., Wang B., Wang R. (2022). Insights into ion-imprinted materials for the recovery of metal ions: Preparation, evaluation and application. Sep. Purif. Technol..

[173] He W., Wu Z., Liu X., Xu H., Jian X., Zhou K., Du J., Wang J., Zhang D. (2025). MOF-based fluorescent hydrogels for rapid and highly selective visual detection of heavy metal ions. Microchem. J..

[174] Cheaburu-Yilmaz C.N., Tanriverdi S.T., Ozer O., Vasile C., Thakur V., Thakur M. (2018). Polysaccharide Containing Gels for Pharmaceutical Applications. Polymer Gels. Gels Horizons: From Science to Smart Materials..

[175] Visan A.I., Negut I. (2025). Sustainable Hydrogels in Water Treatment—A Short Review. Gels.

[176] Peak C.W., Wilker J.J., Schmidt G. (2013). A review on tough and sticky hydrogels. Colloid Polym. Sci..

[177] Alizadehgiashi M., Khuu N., Khabibullin A., Henry A., Tebbe M., Suzuki T., Kumacheva E. (2018). Nanocolloidal Hydrogel for Heavy Metal Scavenging. ACS Nano.

[178] Zhao C., Liu G., Tan Q., Gao M., Chen G., Huang X., Li L., Wang J., Zhang Y., Xu D. (2023). Polysaccharide-based biopolymer hydrogels for heavy metal detection and adsorption. J. Adv. Res..

[179] da Silva Santos D.H., Xiao Y., Chaukura N., Hill J.M., Selvasembian R., Zanta C.L.P.S. (2022). Regeneration of dye-saturated activated carbon through advanced oxidative processes: A review. Heliyon.

[180] Opriș O., Stegarescu A., Lung I., Porav A., Kacso I., Borodi G. (2025). Preparation and Characterization of Materials Based on Graphene Oxide Functionalized with Fe, Mn, Ni, and Cu Oxides and Their Testing for the Removal of Water Pollutants. Materials.

[181] Wang Y., Pan C., Chu W., Vipin A.K., Sun L. (2019). Environmental Remediation Applications of Carbon Nanotubes and Graphene Oxide: Adsorption and Catalysis. Nanomaterials.

[182] Meng L., Shao Z., Li W., Wang J., Hu C., Yang G. (2025). Study on the Mechanism and Modification of Carbon-Based Materials for Pollutant Treatment. Materials.

[183] Bui T.H., Zuverza-Mena N., Dimkpa C.O., Nason S.L., Thomas S., White J.C. (2024). PFAS remediation in soil: An evaluation of carbon-based materials for contaminant sequestration. Environ. Pollut..

[184] Sokovnin S.Y., Gerasimov A.S., Balezin M.E., Ilves V.G. (2025). Investigation of the photocatalytic activity of metal oxide nanoparticles and their derived composites. Nano-Struct. Nano-Objects.

[185] Wojciechowska A., Markowska-Szczupak A., Lendzion-Bieluń Z. (2022). TiO2-Modified Magnetic Nanoparticles (Fe_3_O_4_) with Antibacterial Properties. Materials.

[186] Kubiak A. (2023). Comparative study of TiO_2_–Fe_3_O_4_ photocatalysts synthesized by conventional and microwave methods for metronidazole removal. Sci. Rep..

[187] Shahzad K., Irshad R., Hussain A. (2026). Recent advances in hydrogels for adsorption and electrochemical detection of heavy metals. Macromol. Res..

[188] Fan C. (2026). Interfacing chemistry and materials for heavy metal ion sensing: Mechanistic foundations and adaptive design strategies. Mater. Chem. Front..

[189] Saqib M. (2026). Electrochemical detection of heavy metals using graphene-based sensors: Advances, meta-analysis, toxicity, and sustainable development challenges. Trends Environ. Anal. Chem..

[190] Udawaththa N. (2026). Advances and opportunities in point-of-care detection of toxic metals: A review of electrochemical and non-electrochemical approaches. Analyst.

[191] De Acha N., Elosúa C., Corres J., Arregui F. (2019). Fluorescent sensors for the detection of heavy metal ions in aqueous media. Sensors.

[192] Gogoi N., Barooah M., Majumdar G., Chowdhury D. (2015). Carbon dots rooted agarose hydrogel hybrid platform for optical detection and separation of heavy metal ions. ACS Appl. Mater. Interfaces.

[193] Jiang W., Jin X., Yu X., Wu W., Xu L., Fu F. (2017). Ion-imprinted magnetic nanoparticles for specific separation and concentration of ultra-trace methyl mercury from aqueous sample. J. Chromatogr. A.

[194] Tian Y., Liu J., Qiao J., Ge F., Yang Y., Zhang Q. (2025). Advancements in Electrochemical Sensing Technology for Heavy Metal Ions Detection. Food Chem. X.

[195] Abilkas A., Kazhkenova N., Baptayev B., O’Reilly R.J., Balanay M.P. (2026). Simultaneous Multi-Ion Heavy Metal Sensing Using Pulse and Stripping Voltammetry at Functionalized Nanomaterial-Modified Glassy Carbon Electrodes. Int. J. Mol. Sci..

[196] Lv Y., Zhao Z., Yue H., Deng F., Li H., Xiang C., Wang R., Wang X., Duan Y. (2023). A combined sampling approach for simultaneous detection of volatile organic compounds and heavy metal ions in water using vacuum ultraviolet photoionization time-of-flight mass spectrometry. Int. J. Mass Spectrom..

[197] Alkhadra M.A., Su X., Suss M.E., Tian H., Guyes E.N., Shocron A.N. (2022). Faradaic Deionization of Brackish and Sea Water via Pseudocapacitive Cation and Anion Intercalation into Few-Layered Molybdenum Disulfide. Chem. Rev..

[198] Tauk M., Folaranmi G., Cretin M., Bechelany M., Sistat P., Zhang C., Zaviska F. (2023). Recent advances in capacitive deionization: A comprehensive review on electrode materials. J. Environ. Chem. Eng..

[199] Tang G. (2024). A literature review of electrocoagulation integrated systems on wastewater treatment. Theor. Nat. Sci..

[200] Al-Qodah Z., AL-Rajabi M.M., Da’na E., Al-Shannag M., Bani-Melhem K., Assirey E. (2025). Continuous Electrocoagulation Processes for Industrial Inorganic Pollutants Removal: A Critical Review of Performance and Applications. Water.

[201] de Vargas Brião G., da Costa T.B., Antonelli R., Costa J.M. (2024). Electrochemical processes for the treatment of contaminant-rich wastewater: A comprehensive review. Chemosphere.

[202] Abdi P., Verdijkazemi K., Nejaei A., Takdastan A. (2025). Optimization of ultrasound-electrocatalytic process for improving the quality of biological treatment effluent. Sci. Rep..

[203] Abdollahi J., Alavi Moghaddam M.R., Habibzadeh S. (2023). The role of the current waveform in mitigating passivation and enhancing electrocoagulation performance: A critical review. Chemosphere.

[204] Cheng Z., Jiang X., Cui Z., Jia H., Wang J. (2021). The characteristic of electrode of degradation of bio-electrochemical system based on in-situ ultrasonic monitoring: Biofilm and ion precipitation. Sci. Total Environ..

[205] Conners E.M., Rengasamy K., Bose A. (2022). Electroactive biofilms: How microbial electron transfer enables bioelectrochemical applications. J. Ind. Microbiol. Biotechnol..

[206] Huang H., Ge H., Ren Z., Huang Z., Xu M., Wang X. (2021). Controllable Synthesis of Biocompatible Fluorescent Carbon Dots From Cellulose Hydrogel for the Specific Detection of Hg^2+^. Front. Bioeng. Biotechnol..

[207] Yuan H., Peng J., Ren T., Luo Q., Luo Y., Zhang N., Huang Y., Guo X., Wu Y. (2021). Novel fluorescent lignin-based hydrogel with cellulose nanofibers and carbon dots for highly efficient adsorption and detection of Cr(VI). Sci. Total Environ..

[208] Li C., Wan H., Qiu X., Zhai S., Sun P., Zhao Y. (2025). Multi-dimensional fouling process analysis and anti-fouling strategies for catalytic membranes in long-term water treatment. Water Res..

[209] Shao J., Yu Q., Wang S., Hu Y., Guo Z., Kang K. (2019). Poly(vinyl alcohol)–Carbon Nanodots Fluorescent Hydrogel with Superior Mechanical Properties and Sensitive to Detection of Iron(III) Ions. Macromol. Mater. Eng..

[210] Chan K., Morikawa K., Shibata N., Zinchenko A. (2021). Adsorptive Removal of Heavy Metal Ions, Organic Dyes, and Pharmaceuticals by DNA–Chitosan Hydrogels. Gels.

[211] Fan X., Wang X., Cai Y., Xie H., Han S., Hao C. (2022). Functionalized cotton charcoal/chitosan biomass-based hydrogel for capturing Pb^2+^, Cu^2+^ and MB. J. Hazard. Mater..

[212] Sharma B., Thakur S., Trache D., Yazdani Nezhad H., Thakur V.K. (2020). Microwave-Assisted Rapid Synthesis of Reduced Graphene Oxide-Based Gum Tragacanth Hydrogel Nanocomposite for Heavy Metal Ions Adsorption. Nanomaterials.

[213] Latifah S.A., Nurul A.J., Suhaina I., Ku E.H.K.I., Tuti K.A. (2025). Membrane filtration technologies for sustainable industrial wastewater treatment: A review of heavy metal removal. Desalin. Water Treat..

[214] Sethi S., Medha, Singh G., Sharma R., Kaith B.S., Sharma N. (2022). Fluorescent hydrogel of chitosan and gelatin cross-linked with maleic acid for optical detection of heavy metals. J. Appl. Polym. Sci..

[215] Xu X., Ouyang X.K., Yang L.Y. (2021). Adsorption of Pb(II) from aqueous solutions using crosslinked carboxylated chitosan/carboxylated nanocellulose hydrogel beads. J. Mol. Liq..

[216] Zhao H., Ouyang X.K., Yang L.Y. (2021). Adsorption of lead ions from aqueous solutions by porous cellulose nanofiber–sodium alginate hydrogel beads. J. Mol. Liq..

[217] Li T., Liu X., Li L., Wang Y., Ma P., Chen M. (2019). Polydopamine-functionalized graphene oxide compounded with polyvinyl alcohol/chitosan hydrogels on the recyclable adsorption of Cu(II), Pb(II) and Cd(II) from aqueous solution. J. Polym. Res..

[218] Demirbilek C., Dinç C.O. (2012). Synthesis of diethylaminoethyl dextran hydrogel and its heavy metal ion adsorption characteristics. Carbohydr. Polym..

[219] Godiya C.B., Liang M., Sayed S.M., Li D., Lu X. (2019). Novel alginate/polyethyleneimine hydrogel adsorbent for cascaded removal and utilization of Cu^2+^ and Pb^2+^ ions. J. Environ. Manag..

[220] Sahraei R., Ghaemy M. (2017). Synthesis of modified gum tragacanth/graphene oxide composite hydrogel for heavy metal ions removal and preparation of silver nanocomposite for antibacterial activity. Carbohydr. Polym..

[221] Sahraei R., Sekhavat Pour Z., Ghaemy M. (2017). Novel magnetic bio-sorbent hydrogel beads based on modified gum tragacanth/graphene oxide: Removal of heavy metals and dyes from water. J. Clean. Prod..

[222] Lone S., Yoon D.H., Lee H., Cheong I.W. (2019). Gelatin–chitosan hydrogel particles for efficient removal of Hg(II) from wastewater. Environ. Sci. Water Res. Technol..

[223] Ligarda-Samanez C.A., Huamán-Carrión M.L., Palomino-Rincón H., Taipe-Pardo F., Moscoso-Moscoso E., Cabel-Moscoso D.J., Garcia-Espinoza A.J., Calderón Huamaní D.F., Romero Plasencia J.M., Martinez-Hernandez J.A. (2026). Sustainable Biopolymers for Environmental Applications: Advances and Future Perspectives Toward a Circular Economy. Polymers.

[224] Khim H.C., Bollinger J.-C., Kierczak J. (2025). Pseudo-first-order kinetics in environmental adsorption: Why are there two distinct equations?. Environ. Surf. Interfaces.

[225] Georgieva V.G., Gonsalvesh L., Tavlieva M.P. (2020). Thermodynamics and kinetics of the removal of nickel (II) ions from aqueous solutions by biochar adsorbent made from agro-waste walnut shells. J. Mol. Liq..

[226] Zhang Z., Jiang C., Li D., Lei Y., Yao H., Zhou G., Wang K., Rao Y., Liu W., Xu C. (2020). Micro-mesoporous activated carbon simultaneously possessing large surface area and ultra-high pore volume for efficiently adsorbing various VOCs. Carbon.

[227] Emdadi L., Oh S.C., Wu Y., Oliaee S.N., Diao Y., Zhu G., Liu D. (2016). The role of external acidity of meso-/microporous zeolites in determining selectivity for acid-catalyzed reactions of benzyl alcohol. J. Catal..

[228] Parra M., Gil S., Gaviña P., Costero A.M. (2022). Mesoporous Silica Nanoparticles in Chemical Detection: From Small Species to Large Bio-Molecules. Sensors.

[229] Khan K.S., Alam T., Waheed A., Azim R., Asghar M.A., Palta J.A., Li L., Raza T., Qadir M.F., Manzoor R. (2026). Biochar for soil and wastewater remediation: Heavy metals, organic–inorganic contaminants, and circular economy–driven sustainable agriculture. Farming Syst..

[230] Idrissov N., Bissenova M., Mambetova M., Yeleuov M., Askaruly K., Umirzakov A., Yergaziyeva G., Tsurtsumia O., Serik A., Daulbayev C. (2016). Rice husk–derived activated carbon and SiO2–based materials for dye removal and CO_2_ adsorption. S. Afr. J. Chem. Eng..

[231] Azevedo D.C.S., Araújo J.C.S., Bastos-Neto M., Torres A.E.B., Jaguaribe E.F., Cavalcante C.L. (2007). Microporous activated carbon prepared from coconut shells using chemical activation with zinc chloride. Microporous Mesoporous Mater..

[232] Lansari I., Tizaoui K., Benguella B. (2025). Chitosan-Based Biosorption: A Sustainable Approach for Heavy Metal Removal from Wastewater. Chem. Proc..

[233] Morcillo-Martín R., Espinosa E., Rabasco-Vílchez L., Sanchez L.M., de Haro J., Rodríguez A. (2022). Cellulose Nanofiber-Based Aerogels from Wheat Straw: Influence of Surface Load and Lignin Content on Their Properties and Dye Removal Capacity. Biomolecules.

[234] Parès L., Naasri S., Delattre L., Therriault H., Liberelle B., Crescenzo G.D., Lauzon M.-A., Faucheux N., Paquette B., Virgilio N. (2024). Macroporous chitosan/alginate hydrogels crosslinked with genipin accumulate and retain glioblastoma cancer cells. RSC Adv..

[235] Gibowsky L., Berardinis L.D., Plazzotta S., Manke E., Jung I., Méndez D.A., Heidorn F., Liese G., Husung J., Liese A. (2025). Conversion of natural tissues and food waste into aerogels and their application in oleogelation. Green Chem..

[236] Vipul V.K., Svoboda J., Sedenkova I., Hromadkova J., Galambos M., Tomsik E. (2026). Advanced Pathways for the Preparation of Sensitive Lead(II) Ion Sensor: Ion-Imprinted versus Acid-Assisted Polymerization of 3-Thiopheneacetic Acid with 1-Vinylimidazole Monomers. ACS Omega.

